# Precision Probiotics in Agroecosystems: Multiple Strategies of Native Soil Microbiotas for Conquering the Competitor Ralstonia solanacearum

**DOI:** 10.1128/msystems.01159-21

**Published:** 2022-04-26

**Authors:** Jiakang Yin, Ziliang Zhang, Yixiong Guo, Yue Chen, Yang Xu, Wenxuan Chen, Yanan Shao, Youfeng Yu, Lixia Zhu, Lingling Chen, Lifang Ruan

**Affiliations:** a State Key Laboratory of Agricultural Microbiology, College of Life Science and Technology, Huazhong Agricultural University, Wuhan, People’s Republic of China; b National Key Laboratory of Crop Genetic Improvement, College of Informatics, Huazhong Agricultural University, Wuhan, People’s Republic of China; c Hubei Key Laboratory of Agricultural Bioinformatics, College of Informatics, Huazhong Agricultural University, Wuhan, People’s Republic of China; d College of Humanities and Social Sciences, Huazhong Agricultural University, Wuhan, People’s Republic of China; e The Academy of Agriculture and Forestry Science of Panzhihua City, Panzhihua, People’s Republic of China; Pacific Northwest National Laboratory

**Keywords:** soil microbiome, *Ralstonia solanacearum*, precision probiotics, multifaceted biocontrol

## Abstract

Ralstonia solanacearum (Rs), a soilborne phytopathogen, causes bacterial wilt disease in a broad range of hosts. Common approaches, for example, the direct reduction of the pathogen using classic single broad-spectrum probiotics, suffer from poor colonization efficiency, interference by resident microbiota, and nonnative-microorganism invasion. The soil microbiota plays an important role in plant health. Revealing the intrinsic linkage between the microbiome and the occurrence of disease and then applying it to agroecosystems for the precise control of soilborne diseases should be an effective strategy. Here, we surveyed the differences in the microbiome between healthy and diseased soils used for tomato planting across six climatic regions in China by using 16S rRNA amplicon and metagenomic sequencing. The roles of species associated with disease symptoms were further validated. Healthy soil possessed more diverse bacterial communities and more potential plant probiotics than diseased soil. Healthy soil simultaneously presented multiple strategies, including specifically antagonizing Rs, decreasing the gene expression of the type III secretion system of Rs, and competing for nutrition with Rs. Bacteria enriched in diseased samples promoted the progression of tomato bacterial wilt by strengthening the chemotaxis of pathogens. Therefore, Rs and its collaborators should be jointly combatted for disease suppression. Our research provides integrated insights into a multifaceted strategy for the biocontrol of tomato bacterial wilt based on the individual network of local microbiota.

**IMPORTANCE** In the current work, the relationship between the soil microbiota and tomato bacterial wilt on a large scale offered us a comprehensive understanding of the disease. The delicate strategy of the microbiota in soil used for growing tomatoes to conquer the strong competitor, Rs, was revealed by microbiome research. The collaborators of Rs that coexist in a common niche with Rs strengthened our understanding of the pathogenesis of bacterial wilt. Bacteria enriched in healthy soil that antagonized pathogens with high specificity provide a novel view for ecofriendly probiotics mining. Our study offers new perspectives on soilborne-pathogen biocontrol in agroecosystems by decoding the rule of the natural ecosystem.

## INTRODUCTION

Soilborne phytopathogens infect various crops, leading to high yield losses, and they are hard to control (1–3). As one of the top 10 bacterial phytopathogens, Ralstonia solanacearum (Rs), has extensive geographical distribution (https://gd.eppo.int/taxon/RALSSO/distribution) in China and across the world (4). Rs infects many crops worldwide, including tomato, potato, and tobacco, and inflicts severe damage worldwide (5). Many approaches have been established to alleviate its infectiousness, such as soil amendment and fumigation (6, 7) and breeding of resistant cultivars (8). However, these methods have their respective limitations. Soil amendment and fumigation disturb healthy native microbiota, whereas the breeding of resistant cultivars is restricted by the lack of elite resistant parents and highly diverse *Ralstonia* sp. virulence factors (9). *Bacillus* and Pseudomonas, similar to *Bifidobacterium* species used in the pharmaceutical field, are well-known probiotics used for biocontrol in agroecosystems (10, 11). The therapeutic use of *Bifidobacterium* species has been extensively proven to be effective (12, 13). However, individual variations impair probiotic efficacy (14). Similarly, the single broad-spectrum probiotics used for biocontrol in agroecosystems also encounter setbacks during actual application. For example, Bacillus subtilis exposed to a sucrose-deficient environment has reduced biological control efficiency against soilborne disease (Fusarium wilt) because of its impaired ability to colonize the root (15). Furthermore, introducing nonnative biological control agents that cannot specifically target the pathogen could disrupt the resident microbiota and further result in reduced biodiversity and altered ecosystem functioning (16). Therefore, it is necessary to determine a precise, effective, and safe strategy to control Rs.

The soil microbiome, which interacts intimately with plants, is influenced by many factors, such as plant genotype (17), soil properties (18), climate type (19, 20), and plant health status (21). Recent studies have focused on the soil microbiome and demonstrated its essential roles in plant health and productivity (22–24). Disease-suppressive soil is a promising ecosystem enriched with beneficial microbes that have evolved from the continuous cultivation of a susceptible host plant and can protect the plant from pathogens (24, 25). Many studies have described suppressive soil conquering different pathogens, such as Rhizoctonia solani (24), Fusarium oxysporum (26), and R. solanacearum (27). For example, Carrión et al. identified endophytic root bacteria and functional gene clusters associated with soil that suppress the disease caused by R. solani (28). Beneficial microbes in healthy soil inhibit infection by competing for nutrients, such as iron, essential for pathogens (29) and directly kill pathogens by secreting antibiotics (30) and by generating metabolic chemicals with plant growth-promoting effects and inducing the systemic resistance of plants against pathogens (31, 32). Thus, identifying probiotics enriched in healthy soil microbiota should be an effective method of Rs suppression.

In the present study, we collected 45 healthy and 54 diseased soil samples from 31 tomato-growing areas across six climate types in China. Then, we investigated differentially enriched microbes and their functions in healthy and diseased soil through 16S rRNA amplicon sequencing and metagenomic shotgun sequencing. The roles of certain species associated with disease occurrence were further validated. Our results showed that some helpful microbes, which contributed to plant health through multiple strategies, were enriched in healthy soil. Conversely, microbes enriched in diseased soil accelerated disease progression. Our work provided comprehensive insights into the relationship between microbiomes and bacterial wilt, which can guide the design of a multifaceted and precise strategy to conquer Rs reasonably. Our research could also serve as a reference for the precise control of soilborne pathogens through a microbiome survey.

## RESULTS

### National-scale survey of microbiota in tomato-growing soils in China.

**FIG 1:**
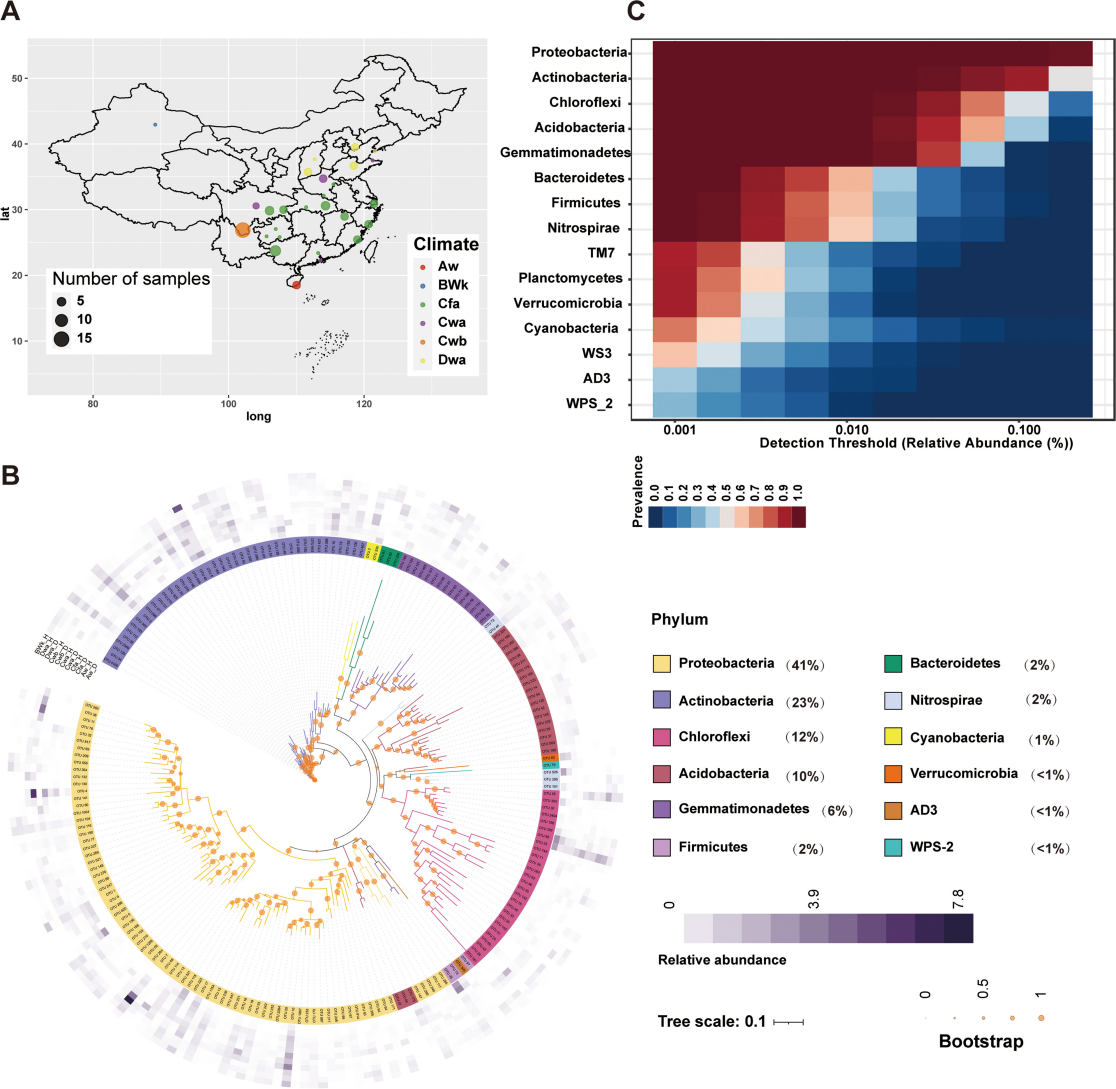
Core microbiome in the soil of major tomato-growing areas in China. (A) Map of the sampling sites. The number of samples of each site is proportional to the circle size. The climate type of the samples is denoted by color. Aw, tropical savanna climate; BWk, cold desert climate; Cfa, humid subtropical climate; Cwa, dry-winter humid subtropical climate; Cwb, dry-winter subtropical highland climate; Dwa, hot summer continental climate. (B) Composition of microbial communities in the six climate types and two health statuses. The innermost layer is the phylogenetic tree of OTUs whose relative abundances are larger than 0.1%. The bootstrap values are proportional to the circle size. The second layer denotes the OTU classifications. The outermost layer shows the relative abundances of OTUs in the six climates and two health statuses (D, diseased; H, healthy). Proportions of main microbes at the phylum level in all samples are shown in parentheses. (C) Heat map of the prevalence of microbial communities at the family level with different detection thresholds (relative abundance).

We collected 99 soil samples with different health statuses from 31 tomato-growing areas across six climate types in China to determine the ecological mechanism by which tomatoes avoided bacterial wilt (Fig. 1A). Fifty-five soil samples were collected from around tomatoes with bacterial wilt caused by Rs. The remaining soil samples were collected from around healthy tomatoes. Table S1 in the supplemental material summarizes this information in detail. All samples were subjected to 16S rRNA amplicon sequencing of the V3-V4 region. A total of 4,716,916 reads were generated as raw data using the Illumina MiSeq platform, and 236,883 unique reads were retained after the removal of reads with short length (<100 bp), low quality (*Q* < 20), and redundancy using USEARCH software (33). We obtained 9,942 operational taxonomic units (OTUs) after clustering the unique reads based on a 97% similarity threshold. The rarefaction curves in Fig. S1 show that no new OTUs could be sampled when more than 70% of the sequences were sampled (Fig. S1). Therefore, sufficient reads were sampled for further analysis.

10.1128/mSystems.01159-21.1 FIG S1 Alpha rarefaction curves for the diseased (*n* = 54) and healthy (*n* = 45) groups. Download FIG S1, TIF file, 0.9 MB. Copyright © 2022 Yin et al. 2022 Yin et al. https://creativecommons.org/licenses/by/4.0/ This content is distributed under the terms of the Creative Commons Attribution 4.0 International license.

10.1128/mSystems.01159-21.7 TABLE S1 Detailed information on samples. Download Table S1, XLSX file, 0.01 MB. Copyright © 2022 Yin et al. 2022 Yin et al. https://creativecommons.org/licenses/by/4.0/ This content is distributed under the terms of the Creative Commons Attribution 4.0 International license.

The microbiotas in all samples comprised 41% *Proteobacteria*, 23% *Actinobacteria*, 12% *Chloroflexi*, 10% *Acidobacteria*, 6% *Gemmatimonadales*, 2% *Bacteroidetes*, 2% *Firmicutes*, 2% *Nitrospirae*, 1% *Cyanobacteria*, and others (Fig. 1B). These bacteria showed different relative abundances in the six climate types and two health statuses (Fig. 1B). Notably, OTU 4, annotated as *Ralstonia*, had higher relative abundance in the diseased soil than in the healthy soil across most climate types (Aw_D, 0.40; Aw_H, 0.09; Cfa_D, 1.31; Cfa_H, 0.55; Cwa_D, 2.58; Cwa_H, 0.02; Cwb_D, 6.29; Cwb_H, 0.46; Dwa_D, 0.02; Dwa_H, 0.01), indicating the reliability of our samples. OTUs 7 and 10 were annotated as Pseudomonas and *Kaistobacter* (family *Sphingomonadaceae*), respectively. Both bacteria showed higher relative abundances in the healthy samples than in the diseased ones (Fig. 1B). Our large-scale sampling across most regions in China enabled us to investigate the core microbiome in the soil of the main tomato-growing areas. *Proteobacteria*, *Actinobacteria*, *Chloroflexi*, *Acidobacteria*, and *Gemmatimonadales* were present in all samples with a 0.01% detection threshold (Fig. 1C), similar to those of members of the global core microbiome (34). Furthermore, their relative abundance in all of the samples was high (∼6% to 41%) (Fig. 1B). Only *Proteobacteria* was detected in all samples when the detection threshold was increased to 0.1% (Fig. 1C). These results showed that some dominant microbes were always present as the core microbiome in the soil of tomato-growing areas in China regardless of health status and climate type. Proteobacteria are abundant and ubiquitous in tomato planting soil, suggesting that they have important ecological attributes. The core microbiome was similar to that observed in a global-scale survey; hence, our results had no sampling bias and highlighted the well-known taxonomic biases of many preexisting culture collections (34).

### Healthy soil possessed more diverse microbiota and more plant probiotics than diseased soil.

**FIG 2:**
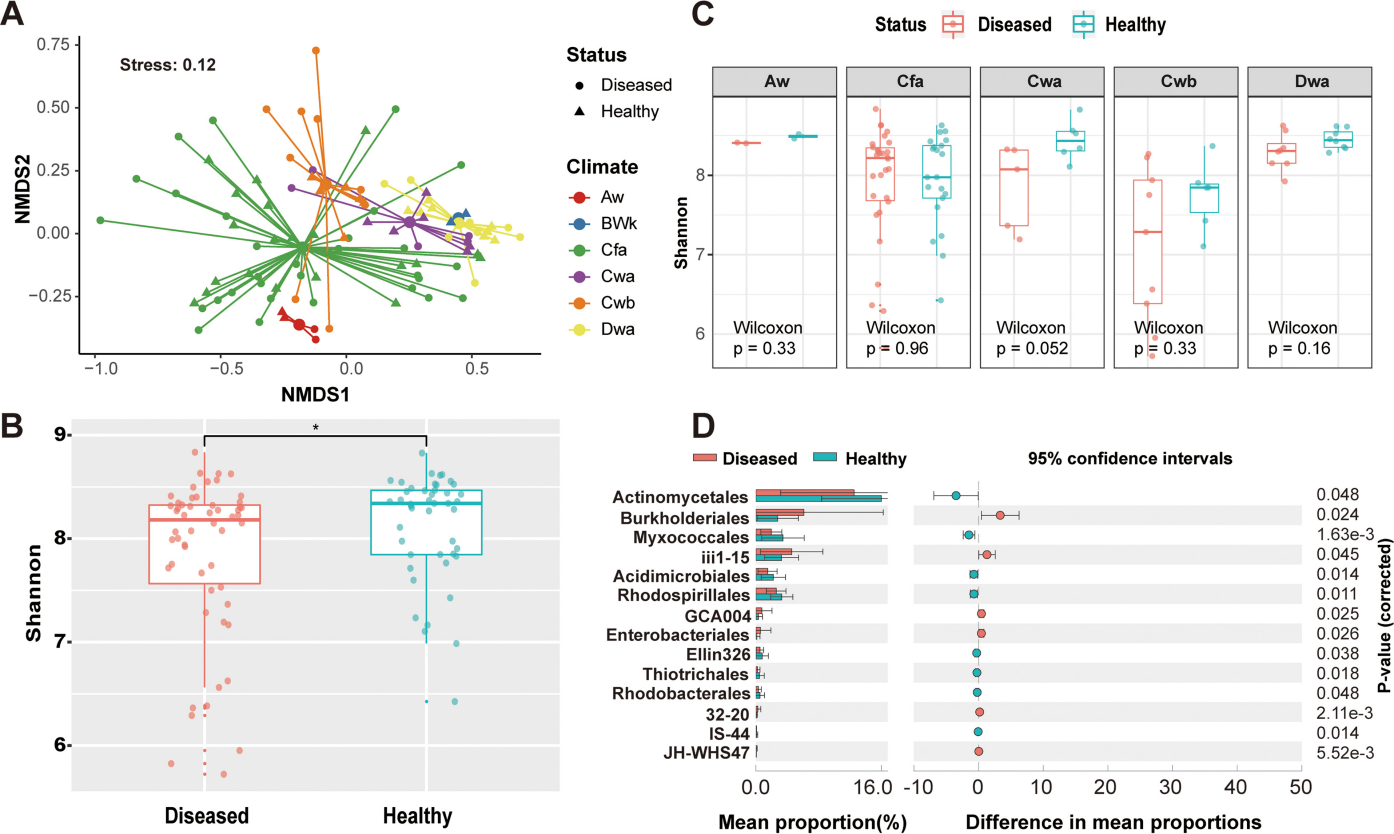
Healthy soil from various climate types has more diversified microbiota and more potential plant probiotics than diseased soil. (A) NMDS analysis of microbial communities in six climate types. Symbol shapes and colors represent the health status and climate types of samples, respectively. (B) Box plot of Shannon alpha diversity index for health status (diseased soil, *n* = 54; healthy soil, *n* = 45). Box-and-whisker plots show high, low, and median values; lower and upper limits denote first and third quartiles, respectively. The significance of the difference between the diseased and healthy groups was tested by the Wilcoxon test. The asterisk indicates a statistically significant difference between treatments (*P* < 0.05). (C) Box plot of Shannon diversity index of healthy and diseased samples from five climate types. The significance of the difference between diseased and healthy groups was tested by the Wilcoxon test. No statistically significant difference was found for any pair of diseased and healthy samples (*P* > 0.05). Box-and-whisker plots show high, low, and median values; lower and upper limits denote first and third quartiles, respectively. Aw, tropical savanna climate; Cfa, humid subtropical climate; Cwa, dry-winter humid subtropical climate; Cwb, dry-winter subtropical highland climate; Dwa, hot summer continental climates. (D) Extended error bar plot based on the relative abundances (percent) of microbes at the order level to explore significant differences between diseased and healthy samples. Corrected *P* values are shown on the right.

We performed nonmetric multidimensional scaling (NMDS) analysis to investigate the separation patterns of the microbiota from all 99 samples. The distribution patterns of the microbiota revealed that the samples were clustered by climate type (Fig. 2A). Permutational multivariate analysis of variance (ANOVA) based on Bray-Curtis dissimilarity also showed that climate type explained a larger proportion (21.3%, *P* = 0.001) than health status (2.4%, *P* = 0.003). Specifically, samples from a dry-winter humid subtropical climate (Cwa), a dry-winter subtropical highland climate (Cwb), and a tropical savanna climate (Aw) were clustered separately. The samples from Aw could be separated based on their health status. However, we could not rule out the relatively small data set in this climate type. Overall, the distribution patterns of the soil microbiota were determined by climate type and health status. Further in-depth analysis was performed to determine the characteristics of healthy soil from the six climate types. The Shannon alpha diversity index was significantly lower in the diseased group than in the healthy one (Wilcoxon test, *P* = 0.038) (Fig. 2B). Similar results were observed in most climate types, although no statistical significance was observed (Wilcoxon test, *P* > 0.05) (Fig. 2C). We conducted a two-sided Welch’s *t* test between the diseased (*n* = 54) and healthy (*n* = 45) samples by using the relative abundances of microbes in each sample to further evaluate the statistically differential microbes in detail. The healthy group was enriched with members of the orders *Actinomycetales*, *Myxococcales*, *Acidimicrobiales*, and *Rhodospirillales* and depleted of members of *Burkholderiales*, iii1-15, and GCA1004 compared with the diseased group (Fig. 2D). Most statistically differential microbes belonged to the core phyla identified above (Fig. 1C); accordingly, tomato health could be determined by core microbes. Taken together, these observations show that healthy soil possessed more diverse microbiota and more potential plant probiotics than diseased soil.

### Potential probiotics with disease-suppressive functions were enriched in healthy soil.

**FIG 3:**
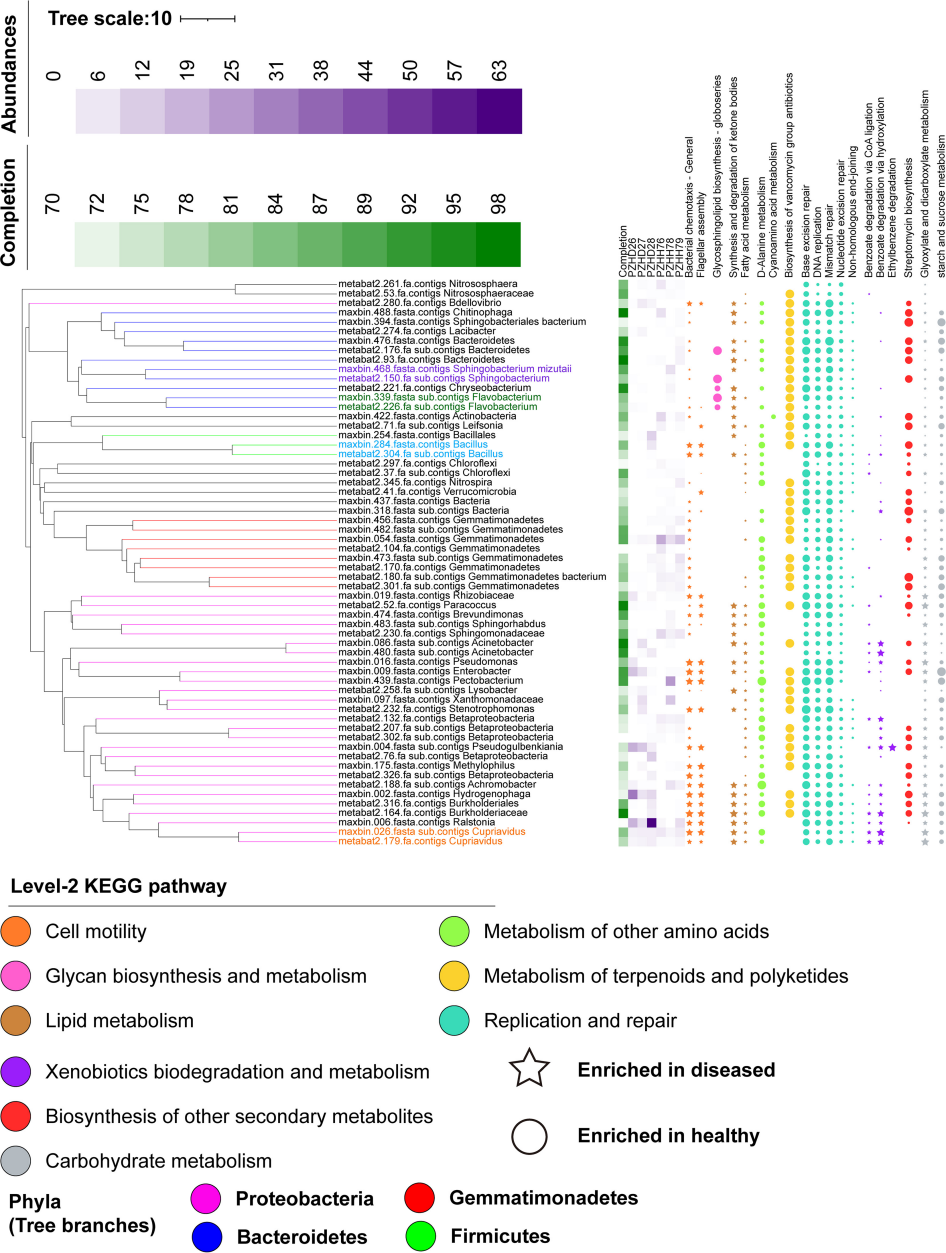
Potential probiotics and their functions are enriched in the healthy group and confer tomato health. The leftmost layer represents the phylogenetic tree of 63 bins with completeness above 70% and contamination below 10%. Branches with different colors represent the four most abundant phyla (*Proteobacteria*, *Bacteriodetes*, *Gemmatimonadetes*, and *Firmicutes*). Labels at the tip of the tree indicate the ID numbers and original taxa of bins. The second layer shows bin completeness. The heat map in the third layer depicts the relative abundance of each bin in each sample. Diseased group: PZHD26, PZHD27, and PZHD28. Healthy group: PZHH76, PZHH78, and PZHH79. Two-sided White’s nonparametric test was performed using STAMP software, and the functions of microorganisms with obvious differences between the diseased and healthy groups are shown in the rightmost layer. Stars and circles indicate functions enriched in the diseased and healthy groups, respectively. The percentage of level 3 KEGG pathways in each bin is proportional to the symbol size. The level 2 KEGG pathway categories of the selected functions are indicated by the color code at the bottom.

We performed metagenomic shotgun sequencing of six representative samples from Renhe District in Panzhihua City (referred to here as PZH; N 26° 49′, E 101° 74′; *n* = 6, including three healthy samples and three diseased samples), which belongs to the Cwb climate type. Our goal was to study the microbial composition at a finer taxonomic level and further investigate the function of the soil microbiota involved in the antagonism against Rs. A total of 4,943,161,732 bp of high-quality reads were generated from all six samples. By profiling the composition of microbiota based on the 16S rRNA extracted from metagenomic shotgun data (Fig. S2A), we found that most sequence reads were assigned to *Bacteria* (93.35%) and that only a minority of reads were assigned to *Archaea* (0.26%), eukaryotes (0.08%), and viruses (0.21%) or were unclassified (6.09%). Consequently, we focused on bacteria and explored the data at the species level, which can provide a more precise resolution. The diseased group was enriched with unclassified *Acidovorax* (order *Burkholderiales*), unclassified Enterobacter (order *Enterobacteriales*), unclassified *Comamonadaceae* (order *Burkholderiales*), and Rs (order *Burkholderiales*); conversely, the healthy group was enriched with unclassified bacteria, unclassified *Gemmatimonadetes*, and unclassified *Proteobacteria* (two-sided White’s nonparametric *t* test, *P* < 0.05) (Fig. S2B and C). We also found similar results from the classification and relative abundance of the bins (Fig. 3). The similarity of these results to those of the 16S amplicon sequencing of national-scale samples (Fig. 1B and 2D) indicated the representativeness of samples from PZH. Additionally, the relative abundance of bacterial wilt phytopathogen Rs was statistically higher (two-sided White’s nonparametric test, *P* = 0.039) in the diseased group than in the healthy group, implying that Rs is the cause of tomato bacterial wilt (Fig. S2C). Principal-component analysis (PCA) revealed that the first axis explained 57.0% of the variance between the two groups (Fig. S2D). Overall, we complemented the results of 16S amplicon sequencing through metagenomic shotgun sequencing.

10.1128/mSystems.01159-21.2 FIG S2 (A) Taxonomic composition of microbiota at the domain level. (B) Taxonomic composition of microbiota at the species level. (A and B) Taxonomic classification was performed based on the 16S rRNA extracted from metagenomic data by using the RDP classifier. (C) Extended error bar plot based on the top 15 microbes in relative abundances (percent) at the species level for exploring significant differences between diseased and healthy samples. Statistical testing was performed using two-sided White’s nonparametric test, and corrected *P* values are shown at right. (D) PCA of the taxa at species level. (E) PCA of the functional categories at the level 3 KEGG pathway. Download FIG S2, TIF file, 2.4 MB. Copyright © 2022 Yin et al. 2022 Yin et al. https://creativecommons.org/licenses/by/4.0/ This content is distributed under the terms of the Creative Commons Attribution 4.0 International license.

Next, we combined taxa and their functions based on their annotations using the Kyoto Encyclopedia of Genes and Genomes (KEGG) to investigate the molecular mechanisms underlying microbes. Our metagenomic data yielded 11,423 KEGG orthologies (KOs), which were regrouped to 247 pathways in the level 3 category. The diseased group was clearly separated from the healthy group as a result of PCA based on the composition of pathways in the level 3 category (Fig. S2E). We found 93 pathways with statistically different abundances (*P* < 0.05) after performing a two-sided White’s nonparametric test between the KEGG pathways of the diseased and healthy groups (Fig. S3). The pathways with obvious abundance differences were selected for a detailed analysis. Among these selected pathways, “Cell motility” (e.g., “Bacterial chemotaxis” and “Flagellar assembly”) was associated with *Ralstonia*, *Cupriavidus*, *Burkholderiaceae*, *Bacillus*, Enterobacter, and *Pseudogulbenkiania*, which were more abundant in the diseased group than in the healthy one (Fig. 3). “Lipid metabolism” (e.g., “Synthesis and degradation of ketone bodies” and “Fatty acid metabolism”), “Xenobiotics biodegradation and metabolism” (e.g., “Benzoate degradation” and “Ethylbenzene degradation”), and “Carbohydrate metabolism” (e.g., “Glyoxylate and dicarboxylate metabolism”) contributed by *Cupriavidus*, *Burkholderiaceae*, *Hydrogenophaga*, *Pseudogulbenkiania*, and Acinetobacter were also enriched in the diseased group (Fig. 3 and Fig. S3). Overall, *Hydrogenophaga* (order *Burkholderiales*), *Pseudogulbenkiania*, and Enterobacter were enriched in the diseased group and showed functional profiles similar to that of Rs (Fig. 3), suggesting that they may act synergistically with Rs for the occurrence of tomato bacterial wilt.

10.1128/mSystems.01159-21.3 FIG S3 Extended error bar plot based on the abundance of functional categories at level 3 KEGG pathway exploring significant differences between diseased and healthy samples. Statistical testing was performed using two-side White’s nonparametric test, and corrected *P* values are shown at right. Download FIG S3, TIF file, 2.7 MB. Copyright © 2022 Yin et al. 2022 Yin et al. https://creativecommons.org/licenses/by/4.0/ This content is distributed under the terms of the Creative Commons Attribution 4.0 International license.

“Glycan biosynthesis and metabolism” (e.g., “Glycosphingolipid biosynthesis–globoseries”), enriched in the healthy group, was attributed to *Bacteroidetes*, *Sphingobacterium*, *Flavobacterium*, and *Chryseobacterium*, which showed relatively higher abundances in the healthy group than in the diseased one (Fig. 3). “Metabolism of other amino acids” (e.g., “d-alanine metabolism” and “Cyanoamino acid metabolism”), enriched in the healthy group, was contributed by almost all taxa identified except *Ralstonia*. *Chitinophaga*, Bacteroidetes, *Sphingobacterium*, *Actinobacteria*, *Bacillus*, and *Gemmatimonadetes* showed higher relative abundances in the healthy group than in the diseased one. They contributed largely to the biosynthesis of antibiotics (e.g., “Biosynthesis of vancomycin group antibiotics” and “Streptomycin biosynthesis”) that possess antimicrobial function. In addition, they contributed to “Carbohydrate metabolism” (e.g., “Starch and sucrose metabolism”), which may compete for available resources with the pathogen, and “Replication and repair” (e.g., “Base excision repair,” “DNA replication,” and “Mismatch repair”), which buffers pathogens through rapid restoration from negative factors (Fig. 3 and Fig. S3) (35, 36). In summary, the healthy group harbored potential probiotics with multiple disease-suppressive functions.

### Suppression of bacterial wilt involved various aspects.

**FIG 4:**
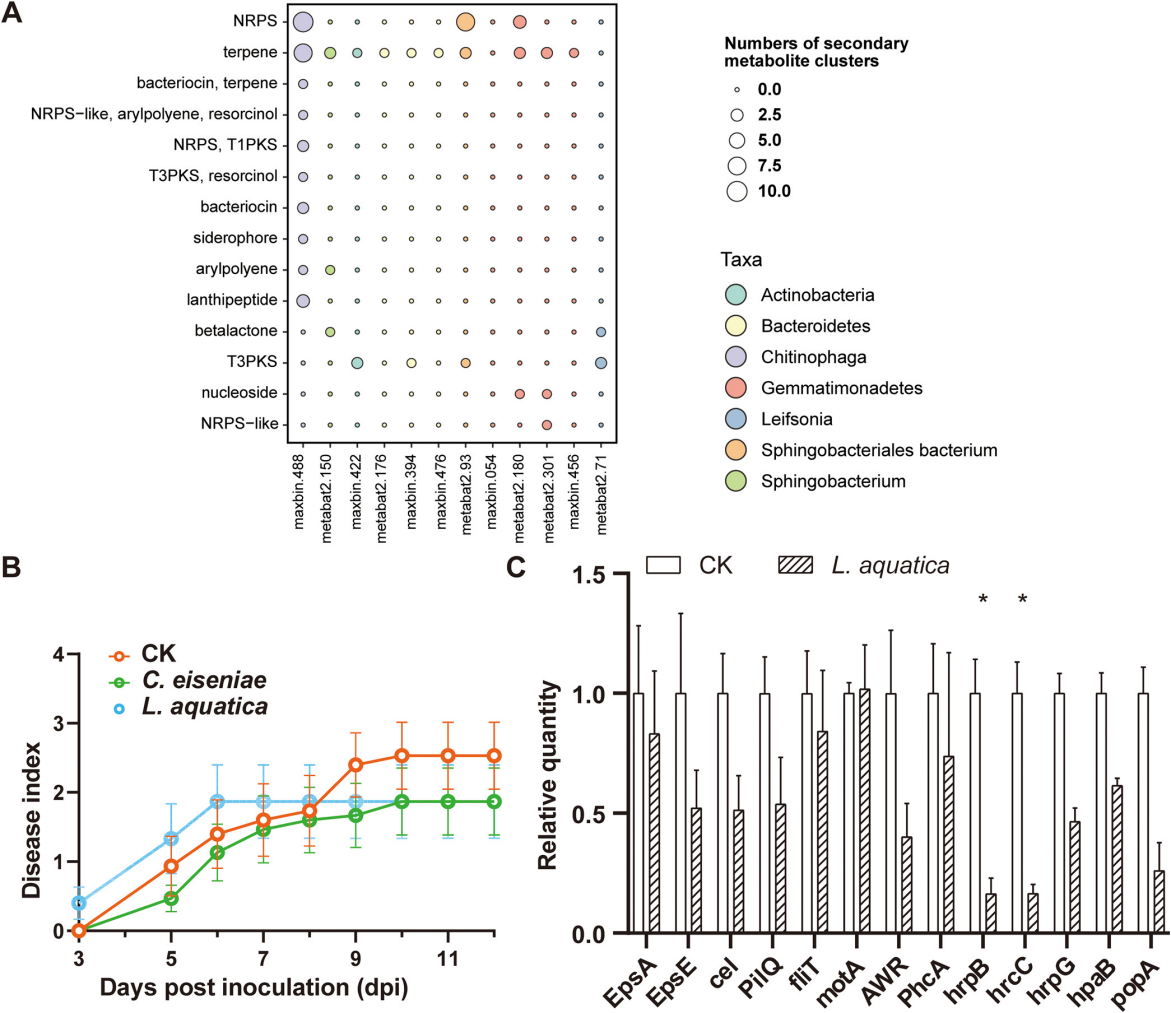
Bacteria enriched in healthy soil suppress bacterial wilt through multiple strategies. (A) Secondary-metabolite analysis of bins enriched in healthy soil. Type and number of biosynthetic gene clusters (BGCs). Taxonomic annotation of bins is denoted by the color on the right. (B) Progression of bacterial wilt on Moneymaker tomatoes preinoculated with potentially beneficial bacteria in sterilized nursery soil. Each dot represents the mean disease index of 15 tomatoes from three independent experiments, and each error bar indicates the standard error of the mean (SEM; *n* = 15). (C) Relative quantification using the quantitative reverse transcription-PCR of disease-related genes of Rs treated with the metabolites of *L. aquatica*. EPS-related genes: *epsA* and *epsE*. Drug-proton antiporter gene: *cel*. Motility-related genes: *pilQ*, *fliT*, and *motA*. T3SS-related genes: *phcA*, *hrpB*, *hrcC*, *hrpG*, *hapB*, *awr*, and *popA*. The relative absolute abundances of these genes were normalized to the 16S rRNA gene. The relative quantity of each gene was normalized to the control. Each bar height indicates the mean abundance of a gene, and each error bar indicates SEM (*n* = 4). The level of significance was calculated using two-way ANOVA with Bonferroni’s correction (*, *P* ≤ 0.05; **, *P* ≤ 0.01; ***, *P* ≤ 0.001).

We selected two representative bins to validate the role of bacteria enriched in healthy soil in protecting tomatoes from bacterial wilt. One was maxbin.488, which possessed the most abundant biosynthesis gene clusters of secondary metabolites with high diversity, including four potentially novel ones (Fig. 4A and Fig. S4B; Table S2). The closest relative found in the Microbial Genome Atlas (MiGA) database (37) was Chitinophaga eiseniae. The other one was metabat2.71, which possessed a high relative abundance of the KEGG pathway “Carbohydrate metabolism” (i.e., “Starch and sucrose metabolism”) (Fig. 3). Its closest relative found in MiGA was Leifsonia aquatica. The zone-of-inhibition assay showed that *C. eiseniae* could antagonize Rs directly while *L. aquatica* could not (Fig. S4A). Interestingly, the bactericidal spectrum experiment including pathogenic and commensal bacteria as targets showed that the *C. eiseniae* exhibited highly specific antagonism toward Rs (Fig. S5).

10.1128/mSystems.01159-21.4 FIG S4 (A) Inhibition zone of *C. eiseniae* and *L. aquatica* against R. solanacearum. Images are representative of three independent experiments. (B) Schematic illustration of the BGCs in maxbin.488. Types of BGCs are indicated on the left with different colors. MIBiG BGC-ID indicating the closest compounds from the MIBiG database is shown on the right. Following is the percentage of genes within the closest known compound that have a significant BLAST hit to genes within the current region. The length of the gene is not to scale. Download FIG S4, TIF file, 0.7 MB. Copyright © 2022 Yin et al. 2022 Yin et al. https://creativecommons.org/licenses/by/4.0/ This content is distributed under the terms of the Creative Commons Attribution 4.0 International license.

10.1128/mSystems.01159-21.5 FIG S5 Inhibition activity of *C. eiseniae* against phytopathogens and commensals. Phytopathogens: A. tumefaciens, Agrobacterium tumefaciens; Pst DC3000, Pseudomonas syringae pv. DC3000; PXO99A, Xanthomonas oryzae pv. oryzae PXO99A; Xcc 8004, Xanthomonas campestris pv. campestris 8004. Commensals: DH5α, Escherichia coli DH5α; *Bacillus*, *Bacillus* sp.; *L. aquatica*, Leifsonia aquatica. Download FIG S5, TIF file, 1.7 MB. Copyright © 2022 Yin et al. 2022 Yin et al. https://creativecommons.org/licenses/by/4.0/ This content is distributed under the terms of the Creative Commons Attribution 4.0 International license.

10.1128/mSystems.01159-21.8 TABLE S2 BGCs of secondary metabolites of maxbin.488. Download Table S2, XLSX file, 0.01 MB. Copyright © 2022 Yin et al. 2022 Yin et al. https://creativecommons.org/licenses/by/4.0/ This content is distributed under the terms of the Creative Commons Attribution 4.0 International license.

Biocontrol assays in pots demonstrated that both potential probiotics could considerably suppress bacterial wilt (Fig. 4B). A high nutritional similarity was also found between *L. aquatica* and Rs (niche overlap index [NOI] = 0.5) and between *C. eiseniae* and Rs (NOI = 0.53) by profiling the utilization of representative carbon resources of the tomato root exudates (38). This finding could provide another example showing that reduced disease severity and similarity in carbon source utilization are positively correlated (39). Additionally, *L. aquatica* showed a strong ability to metabolize sucrose (Fig. S6), which was in accord with the metagenomic analysis (Fig. 3). *C. eiseniae* and *L. aquatica* could also utilize exopolysaccharides (EPSs) such as glucose, mannose, and rhamnose (Fig. S6). Although *L. aquatica* could not antagonize the pathogen, its metabolites reduced the expression of important type III secretion system (T3SS) virulence genes of Rs, such as *hrpB* and *hrcC* (Fig. 4C) (*P* < 0.05).

10.1128/mSystems.01159-21.6 FIG S6 Biolog source utilization profiles of the biological control strains and pathogen-related strains. Strains were clustered based on their ability to utilize sources. White, light blue, and blue indicate the negative, weakly positive, and positive values, respectively. The substrates in red represent the components of the pathogen’s exopolysaccharides. Download FIG S6, TIF file, 0.4 MB. Copyright © 2022 Yin et al. 2022 Yin et al. https://creativecommons.org/licenses/by/4.0/ This content is distributed under the terms of the Creative Commons Attribution 4.0 International license.

In summary, potential probiotics enriched in healthy soil could antagonize pathogens with high specificity, which could guide the development of strategies for the precise control of the pathogen in the agricultural field. Different potential probiotics adopted various tactics to conquer a competitor in the common niche, suggesting natural selection during the interaction among microbiota, plant, and pathogen.

### Species enriched in disease soil accelerate disease progression.

**FIG 5:**
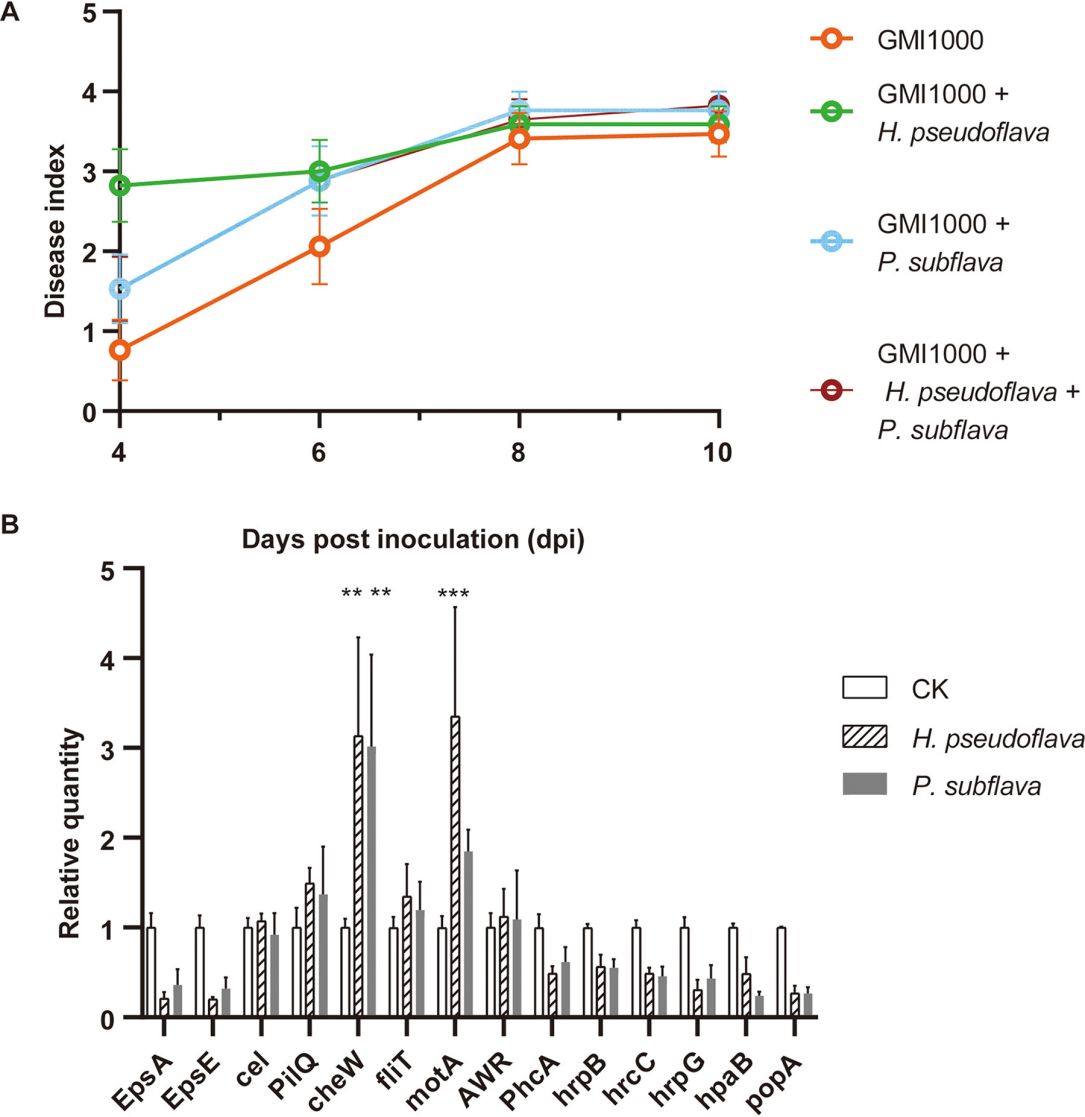
Detrimental species enriched in diseased soil accelerate disease progression. (A) Progression of bacterial wilt on Moneymaker plants coinoculated with potentially detrimental bacteria in sterilized nursery soil. Each dot represents the mean disease index of 17 tomatoes from two independent experiments, and each error bar indicates the SEM (*n* = 17). (B) Relative quantification using quantitative reverse-transcription-PCR of disease-related genes of Rs treated with the metabolites of *H*. *pseudoflava* and *P. subflava*. EPS-related genes: *epsA* and *epsE*. Drug-proton antiporter gene: *cel*. Motility-related genes: *pilQ*, *fliT*, and *motA*. Chemotaxis-related gene: *cheW*. T3SS-related genes: *phcA*, *hrpB*, *hrcC*, *hrpG*, *hapB*, *awr*, and *popA*. The relative absolute abundance of these genes was normalized to the 16S rRNA gene. The relative quantity of each gene was normalized to the control. Each bar height indicates the mean abundance of a gene, and each error bar indicates SEM (*n* = 4). The level of significance was calculated using two-way ANOVA with Bonferroni’s correction (*, *P* ≤ 0.05; **, *P* ≤ 0.01; ***, *P* ≤ 0.001).

We selected two representative bins enriched in diseased soil to understand the role of species enriched in diseased soil (Fig. 3). The closest relatives of maxbin.002 and maxbin.004 found in the MiGA database (37) were Hydrogenophaga pseudoflava and Pseudogulbenkiania subflava, respectively. Coinoculation of *H. pseudoflava* or *P. subflava* with Rs could accelerate the disease progression, and *H. pseudoflava* exerted a stronger influence (Fig. 5A). The metabolites of *H. pseudoflava* significantly increased the expression of the chemotaxis-related gene *cheW* (*P* < 0.01) and the flagellar motor stator gene *motA* (*P* < 0.001) of Rs, and the metabolites of *P. subflava* significantly increased the expression of the *cheW* gene (*P* < 0.01) (Fig. 5B). Rs locates host plants by sensing and chemotaxing toward root exudates using flagellar motility (40). Thus, species enriched in diseased soil might accelerate disease progression by promoting pathogens’ motility and chemotaxis. Therefore, the pathogen and its collaborator should be given attention in the biocontrol process.

## DISCUSSION

A core microbiome is defined as the set of microbes commonly found within a habitat that are hypothesized to provide critical function within the habitat in which they are found (41, 42). For example, a survey of agro-soil microbiota across eastern China revealed that the core microbiota plays vital ecological roles in maintaining belowground multinutrient cycling (43). A nationwide investigation into the core microbiome of tomato planting soil associated with soil health status was conducted in this study. The core microbiome found in the present study is also supposed to be indispensable for regulating soil carbon and nutrient cycling and maintaining the microbial communities in a steady state (44–46). Thus, exploring the core microbiome could reveal the intrinsic functions related to the health status of tomatoes.

In this study, we found that the soil microbiome was mainly shaped by climatic factors. Though other factors like soil physicochemical properties might play an important role in shaping the soil microbiome (47), the climatic factors might be the primary determinant in this process (48). In China, Liang et al. performed a reciprocal transplantation experiment of field soils across a latitudinal gradient to simulate climate change (48). The results showed that climate factors (e.g., mean annual temperature and mean annual precipitation) have a larger effect on the soil bacterial communities than soil attributes (48). In another similar report, researchers found that the transplanted soil microbial community was closer to the microbial community in the local soil than that in the original location over time (49). What is more, when the same soil was transplanted to different climate regions, the microbial community structure was originally the same but was altered significantly in 6 years by soil transplantation (50). In this study, climate type explained a larger proportion of the soil microbial community variation (21.3%, *P* = 0.001) than health status (2.4%, *P* = 0.003), which confirmed the dominant role of climate. Therefore, the climate might be the primary factor in this study.

The diseased group had lower alpha diversity than the healthy group. Decreased alpha diversity is common in microbiomes exposed to adverse circumstances, such as disease and drought (22, 51). Conversely, more diverse microbial communities are often related to the occurrence of more complex and robust networks and a stronger ability to buffer against pathogen invasion (51, 52). A robust community contains multiple species that play similar roles and serve as redundant members, which may explain their ability to buffer disturbance responses. Therefore, restoring the alpha diversity of the microbiome may help extenuate diseases. The healthy group enriched many well-known potential probiotics, such as *Actinomycetales*, *Acidimicrobiales*, and *Myxococcales*. The *Actinomycetales* and *Acidimicrobiales*, which belong to the phylum *Actinobacteria*, are the main sources of natural antimicrobial drug compounds used currently (53). Meanwhile, *Myxococcales* can produce various structurally novel and abundant secondary metabolites and are recognized as another important reservoir of drug-producing microorganisms after *Actinomycetes* (54). Overall, in the tomato-growing areas in China, healthy soil had more diverse microbiotas and more potential probiotics than the diseased soil.

We further conducted metagenomic sequencing of the samples collected from PZH belonging to the Cwb climate type to understand how the soil microbiome contributed to Rs suppression. Based on the finer bacterial composition and functions obtained from the metagenomic analysis, we validated the tomato-protective role of two bacteria enriched in healthy soil (*C. eiseniae* and *L. aquatica*) with different functions in disease suppression and the disease-promoting role of two bacteria enriched in diseased soil (*H. pseudoflava* and *P. subflava*). In order to keep the original state of the microbial community, the soil samples were stored at −80°C as soon as possible before amplicon and metagenomic sequencing. Therefore, the alternative candidates used for biocontrol experiments have to be searched for by genomic similarity. These four strains with high genomic similarity compared to our high-quality bins obtained from the metagenomic analysis were obtained from DSMZ. Although purchasing strains could not restore the native state, this is the optimal alternative strategy so far. Furthermore, the positive results support our strategy to some extent. Our results revealed the high specificity of *C. eiseniae* in antagonizing Rs.

The use of narrow-spectrum antibiotic agents in the agricultural and clinical fields has been advocated because of their ability to reduce negative effects, such as the emergence of resistance and destruction of the beneficial microbiome while antagonizing pathogens (55, 56). For example, thuricin CD, a narrow-spectrum bacteriocin produced by Bacillus thuringiensis, inhibits Clostridioides (formerly Clostridium) difficile in the distal colon without remarkable disruption of the microbiota (57). In addition, CRS3123, a synthetic small-molecule narrow-spectrum antibacterial drug, inhibits C. difficile toxin production and exhibits minimal disruption of normal gut microbiota (58). Therefore, this high specificity could minimize disturbances to the native microbiota and provide precise protection against pathogens. In comparison, *L. aquatica* suppressed the T3SS genes of Rs, and this mechanism implies another promising biocontrol alternative, that is, exerting little selective pressure and low probability of generating bacterial resistance (59). Pathogens deliver type III effectors and manipulate host cellular processes to proliferate in the plant and then cause disease (60). Given the importance of T3SS, *L. aquatica* has ecological implications as well; that is, *L. aquatica* may benefit plants by competing for nutrition (as suggested by the high NOI [0.5] between *L. aquatica* and Rs) from root exudates.

Bacteria such as *H. pseudoflava* and *P. subflava* enriched in diseased soil accelerated disease progression when coinoculated with the pathogen Rs. They increased the expression of Rs’ motility- and chemotaxis-related genes, which may contribute to bacterial wilt at the invasion and colonization stages by responding to specific root exudates and switching their direction of rotation (40, 61). Similarly, synergetic interactions between a seedborne plant-pathogenic bacterium, Burkholderia glumae (Bg), and an airborne plant-pathogenic fungus, Fusarium graminearum (Fg), have been reported (62). Their interactions enable Fg to produce more spores and toxins and assist Bg in achieving aerial dispersal (62). The microbes that collaborate with Rs may benefit from the process of cooperation. For instance, Wang et al. reported that certain bacteria from cow dung help the fungus Arthrobotrys oligospora transition from saprophytic to nematode-predatory form and reduce predation pressure by producing and releasing urea (63). This strategy seems not to be restricted to nematode predation. Burkholderia rhizoxinica, an endosymbiont of a pathogenic fungus belonging to the genus *Rhizopus*, produces rhizoxin to help the fungus cause rice seedling blight (64). The symbiont may also benefit from nutrients from decaying plant materials (64). Therefore, the two species found in the present study may benefit from the process of pathogenesis by utilizing the degradation products of plant cell walls and/or dead plant residues for better proliferation.

**FIG 6:**
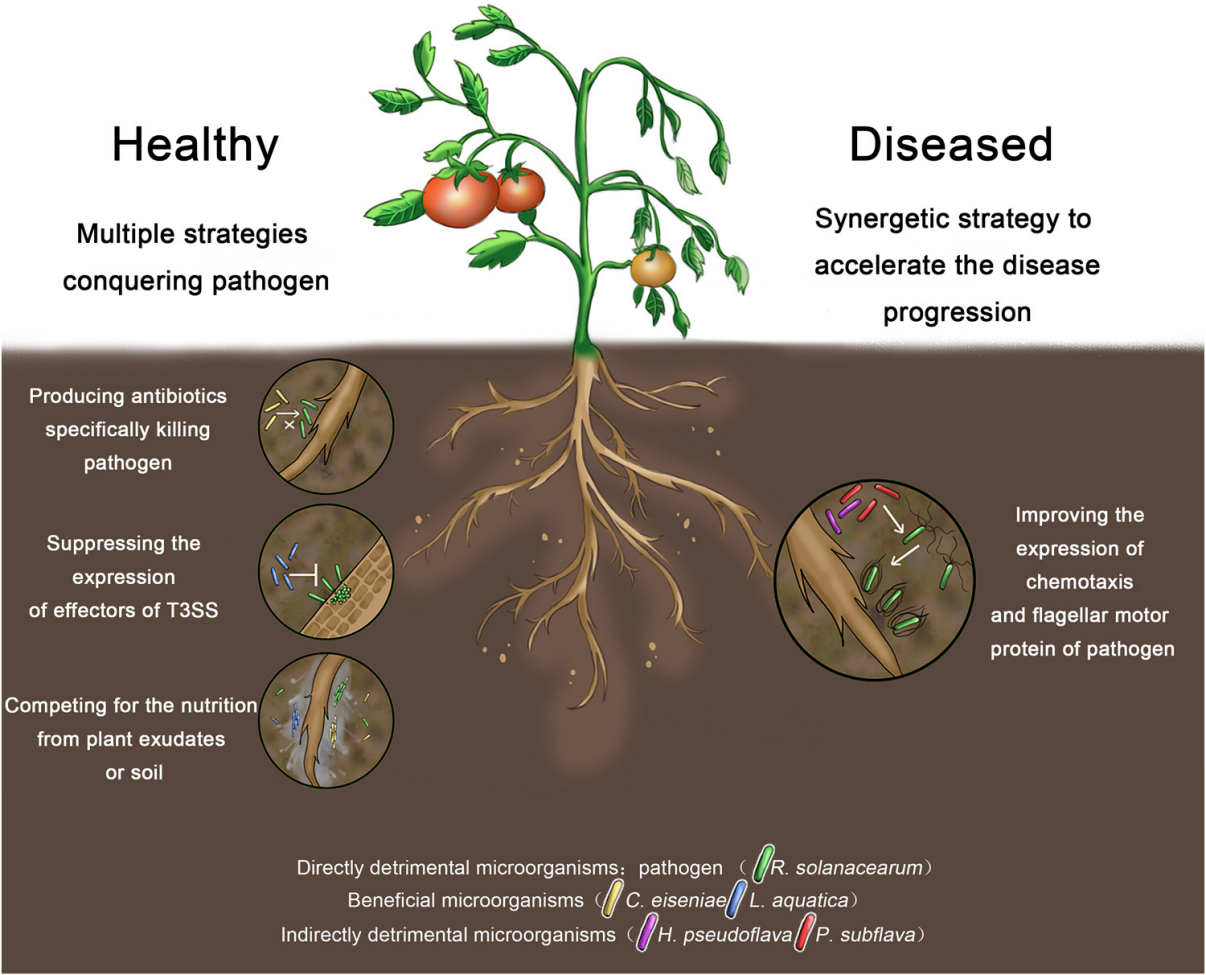
Schematic diagram displaying the intrinsic linkage between the soil microbiome and bacterial wilt of tomato.

In summary, the exploration of beneficial and detrimental microbes and functions associated with the tomato bacterial wilt revealed the intrinsic ecological mechanisms of tomato planting soil (Fig. 6).

## MATERIALS AND METHODS

### Soil sample collection.

After removal of surface components, a sample of the bulk soil close to the tomato roots at a depth of 0 to 10 cm was put into a valve bag and transported back to the laboratory. In all, 99 soil samples with two health statuses were collected from 31 tomato-growing areas across six climate types in China from November 2016 to October 2017. The two health statuses included the diseased and healthy groups. The diseased plants were identified according to the typical green wilt symptom. The healthy soil samples were collected from tomatoes without disease symptoms. Fifty-four of all samples were collected around tomatoes characterized as having bacterial wilt caused by Rs. The remaining soil samples were collected around healthy tomatoes. The sampling sites are shown in Fig. 1A, and detailed information is presented in Table S1. Climate types were inferred according to Köppen climate classification (https://en.wikipedia.org/wiki/K%C3%B6ppen_climate_classification). The six climate types in our study were as follows: Aw, tropical savanna climate; BWk, cold desert climate; Cfa, humid subtropical climate; Cwa, dry-winter humid subtropical climate; Cwb, dry-winter subtropical highland climate; and Dwa, hot summer continental climate. All the samples were stored at −80°C until further analysis.

### 16S rRNA gene V3-V4 amplicon sequences.

Total genomic DNA of samples was extracted using FastDNA spin extraction kits (MP Biomedicals, Santa Ana, CA, USA) at Shanghai Personal Biotechnology Co., Ltd. (Shanghai, China), following the manufacturer’s instructions. The quantity and quality of extracted DNAs were measured using a NanoDrop ND-1000 spectrophotometer (Thermo Fisher Scientific, Waltham, MA, USA) and agarose gel electrophoresis, respectively.

PCR amplification of the bacterial 16S rRNA gene V3-V4 region was performed using the forward primer 338F (5′-ACTCCTACGGGAGGCAGCA-3′) and the reverse primer 806R (5′-GGACTACHVGGGTWTCTAAT-3′). Sample-specific 7-bp barcodes were incorporated into the primers for multiplex sequencing. The PCR mixtures contained 5 μL of Q5 reaction buffer (5×), 5 μL of Q5 high-fidelity GC buffer (5×), 0.25 μL of Q5 high-fidelity DNA polymerase (5 U/μL), 2 μL (2.5 mM) of deoxynucleoside triphosphates (dNTPs), 1 μL (10 μM) of each forward and reverse primer, 2 μL of DNA template, and 8.75 μL of double-distilled water (ddH_2_O). Thermal cycler conditions were as follows: 98°C for 2 min, followed by 25 cycles consisting of denaturation at 98°C for 15 s, annealing at 55°C for 30 s, and extension at 72°C for 30 s, with a final extension of 5 min at 72°C. PCR amplicons were purified with Agencourt AMPure Beads (Beckman Coulter, Indianapolis, IN) and quantified using the PicoGreen double-strand DNA (dsDNA) assay kit (Invitrogen, Carlsbad, CA, USA). After the individual quantification step, amplicons were pooled in equal amounts, and paired-end 2 × 300-bp sequencing was performed using the Illumina MiSeq platform with MiSeq reagent kit v3 at Shanghai Personal Biotechnology Co., Ltd. (Shanghai, China).

### Metagenomic sequencing.

A total of six samples from Renhe District in Panzhihua City (N 26° 49′, E 101° 74′), belonging to the Cwb climate type, were subjected to metagenomic sequencing. Total genomic DNA of samples was extracted at Novogene Bioinformatics Technology Co., Ltd. (Beijing, China), using a magnetic soil and stool DNA kit (Tiangen). DNA concentration was measured using a Qubit dsDNA assay kit in Qubit 2.0 fluorometer (Life Technologies, CA, USA). The optical density (OD) was between 1.8 and 2.0; DNA contents above 1 μg were used to construct the library. A total amount of 1 μg of DNA per sample was used as input material for the DNA sample preparations. Sequencing libraries were generated using a NEBNext Ultra DNA library prep kit for Illumina (New England Biolabs [NEB], USA) following the manufacturer’s recommendations, and index codes were added to attribute sequences to each sample. Briefly, the DNA sample was fragmented by sonication to a size of 350 bp. DNA fragments were end polished, A tailed, and ligated with the full-length adaptor for Illumina sequencing with further PCR amplification. Last, PCR products were purified (AMPure XP system), and libraries were analyzed for size distribution with an Agilent 2100 Bioanalyzer and quantified using real-time PCR. The clustering of the index-coded samples was performed on a cBot cluster generation system according to the manufacturer’s instructions. After cluster generation, the library preparations were sequenced on an Illumina NovaSeq platform (Novogene, China), and paired-end reads were generated.

### Analysis of 16S rRNA gene amplicon sequencing.

Raw data were processed using the QIIME (Quantitative Insights Into Microbial Ecology; v1.9.0) pipeline (65). Briefly, reads that exactly matched the barcodes were assigned to respective samples and identified as valid sequences. Paired-end reads were assembled using the USEARCH (v10) script fastq_mergepairs (33). The low-quality sequences were filtered using USEARCH with the following criteria: sequences that had a length of <100 bp, sequences that had average Phred scores of <20, and sequences that contained ambiguous bases. After chimera detection, the remaining high-quality sequences were clustered into operational taxonomic units (OTUs) at 97% sequence identity by UPARSE (66). A representative sequence was selected from each OTU using default parameters. OTU taxonomic classification was conducted using the Python script assign_taxonomy.py in QIIME (v1.9.0), searching representative sequences set against the Greengenes Database (13_5 release) (67). An OTU table was further generated to record the abundance of each OTU in each sample and the taxonomy of these OTUs. OTUs containing less than 0.1% of the total sequences across all samples were discarded. A phylogenetic tree of retained OTUs was generated by FastTree (68). To minimize the difference of sequencing depth across samples, an averaged, rounded rarefied OTU table was generated by averaging 100 evenly resampled OTU subsets under 90% of the minimum sequencing depth for further analysis.

The OTU-level alpha diversity index (Shannon diversity index) was calculated using the OTU table in QIIME. OTU-level ranked abundance curves were generated to compare the richness and evenness of OTUs among samples. Beta diversity analysis was performed to investigate the structural variation of microbial communities across samples using nonmetric multidimensional scaling (NMDS) analysis with the R package vegan (69). The result was visualized by the R packages ggplot2 (70) and ggalt (71). The core microbiome analysis was performed using a web-based tool MicrobiomeAnalyst (72). Prevalence is the minimum percentage of samples that share this microbe. The detection threshold is the minimum relative abundance of this microbe. The taxonomic compositions and abundances were visualized using the R package ggplot2. Statistical analysis of taxonomic profiles was performed by using STAMP (73). The phylogenetic tree of representative OTUs and OTUs’ relative abundances were visualized using iTOL (74).

### Metagenomic sequences analysis.

Raw data were quality filtered using fastp (75) as follows: paired reads were removed when the number of N in any sequencing read exceeded 10% of the bases and when the number of low-quality bases (*Q* ≤ 5) in any sequencing read exceeded 50% of the bases. We used FastQ Screen (v0.14.0) (76) to detect read contamination originating from tomato (Solanum lycopersicum). There was hardly any contamination from the tomato genome (data not shown); therefore, we ignored the step of filtering host genome reads. In total, we obtained 4,943,161,732 bp of clean data for further analysis by using SqueezeMeta v1.1.0 in coassembly mode (77). In detail, reads from all samples were pooled and a single assembly was done using Megahit with default arguments (78). By performing contig statistics using prinseq (79), a total of 6,682,021 contigs were generated: the minimum length was 200 bp, the maximum length was 1,091,085 bp, the average length was 739 bp, and *N*_50_ was 851 bp. RNAs were predicted using Barrnap (https://github.com/tseemann/barrnap), and 16S rRNA sequences were taxonomically classified using the RDP classifier (80). Open reading frames (ORFs) were predicted using Prodigal (81). Similarity searches in GenBank (82) and KEGG (83) were done using Diamond (84). Read mapping against contigs was performed using Bowtie2 (85). Binning was done using MaxBin2 (86) and Metabat2 (87). Then, the binning results were combined using DAS Tool (88). Bin statistics were computed using CheckM (89). Pathway prediction for KEGG (83) databases was done using MinPath (90).

Taxonomy visualization was done with the R package SQMtools included in the SqueezeMeta pipeline. KOs were regrouped to pathways at level 3 KEGG category using the script pathway_pipeline.py included in the PICRUSt2 pipeline (91). Statistical analysis of taxonomic and level 3 KEGG functional profiles was performed with STAMP (73). Principal-component analysis (PCA) was conducted with the R package ggbiplot (https://github.com/vqv/ggbiplot). We used sqm2itol.pl, which is included in the SqueezeMeta pipeline, to select statistically differential functions and transform results from SqueezeMeta to files needed by iTOL. Amino acid identity across bins that had at least 70% completeness and at most 10% contamination was calculated by compareM (https://github.com/dparks1134/CompareM). The taxonomic assignment of bins was assigned based on the last-common-ancestor (LCA) algorithm. Finally, the phylogenetic tree of the 63 bins retained, the relative abundances of bins, and the abundance of selected functions in each bin were visualized using iTOL (74).

### Secondary metabolites analysis of the bins enriched in the healthy group.

To explore the biosynthetic gene clusters (BGCs) of the secondary metabolites in detail, we analyzed the bins enriched in the healthy group and displayed a high relative abundance of the KEGG pathway “Biosynthesis of other secondary metabolites” (Fig. 3) using antiSmash v5.1.2 (92). These bins consist of maxbin.054, maxbin.394, maxbin.422, maxbin.456, maxbin.476, maxbin.488, metabat2.150, metabat2.176, metabat2.180, metabat2.301, metabat2.71, and metabat2.93. The FASTA files of these bins were analyzed with the following parameters: –taxon bacteria, –fullhmmer, –smcon-trees, –cb-knownclusters, –cb-general, –pfam2go, and –genefinding-tool prodigal. Then the type and number of BGCs were counted manually and visualized using ggplot2 (70). The taxonomic information for the bins was presented according to the results of the metagenomic analysis. The BGCs structure of maxbin.488 was plotted using Adobe Illustrator (93).

### Inhibition assays.

To validate the potential biocontrol effect of the bins enriched in the healthy group on the suppression of bacterial wilt, we selected two bins. The prerequisites were higher relative abundance in the healthy group and high completion of the draft genome for precise taxonomic classification. One is maxbin.488 (completeness, 98.06%), possessing the most diverse and abundant BGCs, including four potentially novel BGCs (Fig. 4B); its closest relative found by MiGA (37) in the database was Chitinophaga eiseniae (average amino acid identity [AAI], 94.20%). The other is metabat2.71 (completeness, 79.98%), which possesses a high relative abundance of the KEGG pathway “Carbohydrate metabolism” (i.e., “Starch and sucrose metabolism”) (Fig. 3), and its closest relative found by MiGA was Leifsonia aquatica (AAI, 69.22%). It is reported that several primary metabolic pathways, like sucrose uptake and catabolism, are highly expressed during pathogenesis in Rs (94). The virulence of a mutant with deficiencies in sucrose uptake and catabolism was significantly reduced (94).

The two strains, *C. eiseniae* (DSM 22224) and *L. aquatica* (DSM 20146), were purchased from the DSMZ (Braunschweig, Germany). They were originally isolated from vermicompost and distilled water, respectively. We first investigated the antagonistic activity of these two strains against Rs strain GMI1000 (race 1, biovar 3, phylotype I) using the zone-of-inhibition assay. *C. eiseniae*, *L. aquatica*, and Rs GMI1000 were streaked on R2A agar medium (95), *Corynebacterium* agar medium (casein peptone, tryptic digest, 10.0 g; yeast extract, 5.0 g; glucose, 5.0 g; NaCl, 5.0 g; agar, 15 g; distilled water, 1,000 mL; pH 7.2 to 7.4), and NB agar medium (hipolypepton, [trade name] 5.0 g; yeast extract, 1.0 g; beef extract, 3.0 g; sucrose, 10 g; agar, 15 g; distilled water, 1,000 mL; pH 7.0) at 28°C for single colonies, respectively. Colonies were inoculated into their corresponding liquid growth medium for propagation overnight with shaking (180 rpm). Then, 100 μL of GMI1000 culture was spread onto NB agar medium, and 100-μL cultures of tested strains were added into the holes that had been drilled by 1-mL pipette tips.

To evaluate the antagonistic specificity of *C. eiseniae* on R. solanacearum, we conducted inhibition assays on common phytopathogens, including Agrobacterium tumefaciens, Pseudomonas syringae pv. DC3000, Xanthomonas oryzae pv. oryzae PXO99A, and Xanthomonas campestris pv. campestris 8004, and commensals, including *Bacillus* sp., *L. aquatica*, and Escherichia coli DH5α. All of the bacteria are stored in our lab. They were streaked on NB agar medium, and single colonies were inoculated into liquid NB medium for propagation overnight with shaking at 28°C. Then, the following steps were as described above. A liquid medium was used as a negative control. Each treatment contained at least three holes. Experiments were replicated at least three times. All the plates were incubated at 28°C for 24 to 48 h, and the inhibition zones on the plates were recorded.

### Pot experiments in the greenhouse.

Next, we investigated the suppression effect on bacterial wilt using the well-known commercial cultivar Moneymaker, which is susceptible to Rs. Tomato seeds were germinated on a plate for 7 days at room temperature and transferred into about 100 g sterile nursery soil. Plants were grown in a greenhouse with a diurnal cycle of 12 h day/12 h night at 28 ± 2°C and were watered when needed with tap water. Strains to be tested and GMI1000 were prepared as bacterial suspensions by resuspending the pellet centrifuged from cultures with sterile water. The optical density (OD) was adjusted to 1.0 at 600 nm. About 20 days after transplanting, 20-mL culture suspensions of strains to be tested were poured onto each tomato to soak the base of the plant, resulting in ∼10^8^ CFU/g soil. Tomatoes inoculated with sterile water were used as positive controls. Five days after inoculation, 20-mL culture suspensions of GMI1000 were inoculated per tomato by a soil-drenching method as described above (∼10^8^ CFU/g soil). Each treatment consisted of five tomato plants. Experiments were replicated three times.

To understand the roles that species enriched in diseased soils played in disease progression, we selected two representative bins that were enriched in the diseased soil and showed functional profiles similar to that of the pathogen (Fig. 3). They were maxbin.002 (completeness, 72.05%) and maxbin.004 (completeness, 75.64%), whose closest relatives found by MiGA (37) in the database were Hydrogenophaga pseudoflava (AAI, 98.21%) and Pseudogulbenkiania subflava (AAI, 95.14%), respectively. They were purchased from the DSMZ. They were originally isolated from water and a cold spring, respectively. *H. pseudoflava* (DSM 1034) was cultured in NA (yeast extract, 0.5 g; beef extract, 3.0 g; glucose, 10 g; peptone, 5 g; distilled water, 1,000 mL; pH 7.0) liquid medium, and *P. subflava* (DSM 22618) was cultured in R2A liquid medium. The bacterial suspensions were prepared and inoculated as described above. The inoculations were split into four groups: 20 mL of pathogen suspensions alone, 10 mL of *H. pseudoflava* suspensions with 10 mL of pathogen suspensions, 10 mL of *P. subflava* suspensions with 10 mL of pathogen suspensions, and 6.7 mL of each of the three suspensions (∼10^8^ CFU/g soil). Each treatment consisted of 7 to 10 tomato plants. Experiments were replicated twice.

Seedling plates were rearranged randomly every 2 days, and disease progression was monitored for up to 12 days. Disease progression was recorded using the following disease index: 0, no disease symptoms; 1, 1 to 25% of leaves wilted; 2, 26 to 50% of leaves wilted; 3, 51 to 75% of leaves wilted; 4, 76 to 100% of leaves wilted (96). Disease incidence (DI; in percent) was calculated as follows: {∑[(number of diseased plants × disease index)/(total number of plants × highest disease index)]} × 100. Biological control efficacy (BE; in percent) was calculated as follows: [(disease index of control − disease index of treatment group)/disease index of control] × 100. Statistical analysis was performed using GraphPad Prism v8.0.

### RT-qPCR.

Rs was cultured in modified BMM (97) liquid medium [KH_2_PO_4_, 13.6 g; (NH_4_)_2_SO_4_, 2 g; L-glutamic acid, 2.9 g; glucose, 0.18 g; FeSO_4_·7H_2_O, 0.5 mg; pH, 6.5] at 28°C with shaking to reach an OD of 0.7 at 600 nm. Culture suspensions of *L. aquatica*, *H. pseudoflava*, and *P. subflava* growing in their corresponding liquid media (NB, NA, and R2A, respectively) were centrifuged at 8,000 rpm for 1 min, and the supernatant was filtered using a 0.22-μm microfiltration membrane. Then, 100 μL of the filtered supernatant was added to 1-mL suspensions of Rs, and suspensions without added supernatant were used as controls. One hour later, 1-mL suspensions were centrifuged, and the pellet was collected for RNA extraction, which was performed using a TransZol Up Plus RNA kit (Transgene catalog no. ER501-01) following the manufacture’s instructions. Genomic DNA contamination was eliminated by treating the samples with gDNA Eraser (TaKaRa catalog no. RR047A-1) for 2 min at 42°C. Reverse transcription was conducted using a PrimeScript RT reagent kit (TaKaRa catalog no. RR047A). The quality of cDNA was tested using the *fliT* gene. Quantitative reverse transcription PCR (RT-qPCR) mixtures contained 5 μL of SYBR qPCR master mix (5×), 3 μL of ddH_2_O, 0.5 μL (10 μM) of each forward and reverse primer, and 1 μL of cDNA template. Thermal cycler conditions were as follows: 95°C for 3 min, followed by 40 cycles consisting of denaturation at 95°C for 15 s, annealing at 56°C for 20 s, and extension at 72°C for 20 s. Exopolysaccharides-related genes included *epsA* and *epsE*. The drug-proton antiporter gene was *cel*. Motility-related genes included *pilQ*, *fliT*, and *motA*. The chemotaxis-related gene was *cheW*. T3SS-related genes included *phcA*, *hrpB*, *hrcC*, *hrpG*, *hapB*, *awr*, and *popA*. Primers were either adopted from the work of Song (98) or designed using Clone Manager v8, and they are listed in Table S3. Data were analyzed using QuantStudio real-time PCR software v1.3 using ΔΔ*C_T_* for relative quantification. The relative absolute abundance of these genes was normalized to the 16S rRNA gene. The relative quantity of each gene was normalized to the control. Two-way ANOVA with Bonferroni’s correction was performed for multiple comparisons in GraphPad Prism 8.0. Each treatment had two biological repeats and three technological repeats. Each experiment was repeated twice.

10.1128/mSystems.01159-21.9 TABLE S3 Primers for RT-qPCR. Download Table S3, XLSX file, 0.01 MB. Copyright © 2022 Yin et al. 2022 Yin et al. https://creativecommons.org/licenses/by/4.0/ This content is distributed under the terms of the Creative Commons Attribution 4.0 International license.

### Determination of carbon utilization profiles.

The similarity in carbon source utilization is significant in determining the ability to suppress disease (39). To investigate whether this aspect was important for disease suppression in this study, carbon utilization profiles of the representative bacteria were characterized by Biolog (GNIII MicroPlates). *C. eiseniae* and *P. subflava* were streaked on R2A agar plates, *L. aquatica* and Rs GMI1000 were streaked on NB agar plates, and *H. pseudoflava* was streaked on NA agar plates at 28°C for single colonies; then, procedures were performed according to the manufacture’s instructions. Bacterial colonies were inoculated into the inoculating fluid (IF) to a final density of 90 to 98% T (turbidity). Then, 100 μL of cell suspension was added to each GENIII MicroPlate well, and the plate was incubated at 28°C for 24 to 48 h. OD was measured using the OmniLog system. The values were blank-corrected and normalized by dividing each value with the highest value, resulting in final values between 0 and 1. Here, we defined the values as the utilization index, which was considered positive at a value of ≥0.4, weakly positive from 0.4 to 0.2, and negative at <0.2. The nutritional similarity (in percent) was calculated using the NOI, adapted from the work of Ji and Wilson (39), as follows: (number of carbon sources used by the two testing strains/higher value of the number of carbon sources used by the two testing strains) × 100.

### Data availability.

All the raw sequence data have been deposited in the Sequence Read Archive database of NCBI under the accession number PRJNA597176.

## References

[1] Martins PM, Merfa MV, Takita MA, De Souza AA. 2018. Persistence in phytopathogenic bacteria: do we know enough? Front Microbiol 9:1099. doi:10.3389/fmicb.2018.01099. 29887856 PMC5981161

[2] Kennelly MM, Cazorla FM, de Vicente A, Ramos C, Sundin GW. 2007. *Pseudomonas syringae* diseases of fruit trees: progress toward understanding and control. Plant Dis 91:4–17. doi:10.1094/PD-91-0004. 30781059

[3] Michielse CB, Rep M. 2009. Pathogen profile update: *Fusarium oxysporum*. Mol Plant Pathol 10:311–324. doi:10.1111/j.1364-3703.2009.00538.x. 19400835 PMC6640313

[4] Mansfield J, Genin S, Magori S, Citovsky V, Sriariyanum M, Ronald P, Dow M, Verdier V, Beer SV, Machado MA, Toth I, Salmond G, Foster GD. 2012. Top 10 plant pathogenic bacteria in molecular plant pathology. Mol Plant Pathol 13:614–629. doi:10.1111/j.1364-3703.2012.00804.x. 22672649 PMC6638704

[5] Hayward AC. 1991. Biology and epidemiology of bacterial wilt caused by *Pseudomonas solanacearum*. Annu Rev Phytopathol 29:65–87. doi:10.1146/annurev.py.29.090191.000433. 18479193

[6] Mao L, Jiang H, Wang Q, Yan D, Cao A. 2017. Efficacy of soil fumigation with dazomet for controlling ginger bacterial wilt (*Ralstonia solanacearum*) in China. Crop Prot 100:111–116. doi:10.1016/j.cropro.2017.06.013.

[7] Michel VV, Mew T. 1998. Effect of a soil amendment on the survival of *Ralstonia solanacearum* in different soils. Phytopathology 88:300–305. doi:10.1094/PHYTO.1998.88.4.300. 18944952

[8] Huet G. 2014. Breeding for resistances to *Ralstonia solanacearum*. Front Plant Sci 5:715. doi:10.3389/fpls.2014.00715. 25566289 PMC4264415

[9] Boshou L. 2005. A broad review and perspective on breeding for resistance to bacterial wilt, p 225–238. *In* Allen C, Prior P, Hayward AC (ed), Bacterial wilt disease and the Ralstonia solanacearum species complex. APS Press, St. Paul, MN.

[10] Cantoro R, Palazzini JM, Yerkovich N, Miralles DJ, Chulze SN. 2021. *Bacillus velezensis* RC 218 as a biocontrol agent against *Fusarium graminearum*: effect on penetration, growth and TRI5 expression in wheat spikes. BioControl 66:259–270. doi:10.1007/s10526-020-10062-7.

[11] Zhang Q, Kong X, Li S, Chen XJ, Chen XJ. 2020. Antibiotics of *Pseudomonas protegens* FD6 are essential for biocontrol activity. Australas Plant Pathol 49:307–317. doi:10.1007/s13313-020-00696-7.

[12] Din AU, Hassan A, Zhu Y, Zhang K, Wang Y, Li T, Wang Y, Wang G. 2020. Inhibitory effect of *Bifidobacterium bifidum* ATCC 29521 on colitis and its mechanism. J Nutr Biochem 79:108353. doi:10.1016/j.jnutbio.2020.108353. 32145470

[13] Pinto-Sanchez MI, Hall GB, Ghajar K, Nardelli A, Bolino C, Lau JT, Martin F-P, Cominetti O, Welsh C, Rieder A, Traynor J, Gregory C, De Palma G, Pigrau M, Ford AC, Macri J, Berger B, Bergonzelli G, Surette MG, Collins SM, Moayyedi P, Bercik P. 2017. Probiotic *Bifidobacterium longum* NCC3001 reduces depression scores and alters brain activity: a pilot study in patients with irritable bowel syndrome. Gastroenterology 153:448–459.E8. doi:10.1053/j.gastro.2017.05.003. 28483500

[14] Veiga P, Suez J, Derrien M, Elinav E. 2020. Moving from probiotics to precision probiotics. Nat Microbiol 5:878–880. doi:10.1038/s41564-020-0721-1. 32393856

[15] Tian T, Sun B, Shi H, Gao T, He Y, Li Y, Liu Y, Li X, Zhang L, Li S, Wang Q, Chai Y. 2021. Sucrose triggers a novel signaling cascade promoting *Bacillus subtilis* rhizosphere colonization. ISME J 15:2723–2737. doi:10.1038/s41396-021-00966-2. 33772107 PMC8397739

[16] Traveset A, Richardson DM. 2014. Mutualistic interactions and biological invasions. Annu Rev Ecol Evol Syst 45:89–113. doi:10.1146/annurev-ecolsys-120213-091857.

[17] Santos-Medellín C, Edwards J, Liechty Z, Nguyen B, Sundaresan V. 2017. Drought stress results in a compartment-specific restructuring of the rice root-associated microbiomes. mBio 8:e00764-17. doi:10.1128/mBio.00764-17. 28720730 PMC5516253

[18] Mapelli F, Marasco R, Fusi M, Scaglia B, Tsiamis G, Rolli E, Fodelianakis S, Bourtzis K, Ventura S, Tambone F, Adani F, Borin S, Daffonchio D. 2018. The stage of soil development modulates rhizosphere effect along a High Arctic desert chronosequence. ISME J 12:1188–1198. doi:10.1038/s41396-017-0026-4. 29335640 PMC5931989

[19] Zhou J, Deng Y, Shen L, Wen C, Yan Q, Ning D, Qin Y, Xue K, Wu L, He Z, Voordeckers JW, Nostrand JDV, Buzzard V, Michaletz ST, Enquist BJ, Weiser MD, Kaspari M, Waide R, Yang Y, Brown JH. 2016. Temperature mediates continental-scale diversity of microbes in forest soils. Nat Commun 7:12083. doi:10.1038/ncomms12083. 27377774 PMC4935970

[20] Maestre FT, Delgado-Baquerizo M, Jeffries TC, Eldridge DJ, Ochoa V, Gozalo B, Quero JL, García-Gómez M, Gallardo A, Ulrich W, Bowker MA, Arredondo T, Barraza-Zepeda C, Bran D, Florentino A, Gaitán J, Gutiérrez JR, Huber-Sannwald E, Jankju M, Mau RL, Miriti M, Naseri K, Ospina A, Stavi I, Wang D, Woods NN, Yuan X, Zaady E, Singh BK. 2015. Increasing aridity reduces soil microbial diversity and abundance in global drylands. Proc Natl Acad Sci USA 112:15684–15689. doi:10.1073/pnas.1516684112. 26647180 PMC4697385

[21] Zhang Y, Xu J, Riera N, Jin T, Li J, Wang N. 2017. Huanglongbing impairs the rhizosphere-to-rhizoplane enrichment process of the citrus root-associated microbiome. Microbiome 5:97. doi:10.1186/s40168-017-0304-4. 28797279 PMC5553657

[22] Xu L, Naylor D, Dong Z, Simmons T, Pierroz G, Hixson KK, Kim YM, Zink EM, Engbrecht KM, Wang Y. 2018. Drought delays development of the sorghum root microbiome and enriches for monoderm bacteria. Proc Natl Acad Sci USA 115:E4284–E4293. doi:10.1073/pnas.1717308115. 29666229 PMC5939072

[23] Berendsen RL, Vismans G, Yu K, Song Y, de Jonge R, Burgman WP, Burmølle M, Herschend J, Bakker PA, Pieterse CM. 2018. Disease-induced assemblage of a plant-beneficial bacterial consortium. ISME J 12:1496–1507. doi:10.1038/s41396-018-0093-1. 29520025 PMC5956071

[24] Mendes R, Kruijt M, de Bruijn I, Dekkers E, van der Voort M, Schneider JHM, Piceno YM, DeSantis TZ, Andersen GL, Bakker PAHM, Raaijmakers JM. 2011. Deciphering the rhizosphere microbiome for disease-suppressive bacteria. Science 332:1097–1100. doi:10.1126/science.1203980. 21551032

[25] Raaijmakers JM, Mazzola M. 2016. Soil immune responses. Science 352:1392–1393. doi:10.1126/science.aaf3252. 27313024

[26] Cha J-Y, Han S, Hong H-J, Cho H, Kim D, Kwon Y, Kwon S-K, Crüsemann M, Bok Lee Y, Kim JF, Giaever G, Nislow C, Moore BS, Thomashow LS, Weller DM, Kwak Y-S. 2016. Microbial and biochemical basis of a Fusarium wilt-suppressive soil. ISME J 10:119–129. doi:10.1038/ismej.2015.95. 26057845 PMC4681868

[27] Lee S-M, Kong HG, Song GC, Ryu C-M. 2021. Disruption of Firmicutes and Actinobacteria abundance in tomato rhizosphere causes the incidence of bacterial wilt disease. ISME J 15:330–347. doi:10.1038/s41396-020-00785-x. 33028974 PMC7852523

[28] Carrión VJ, Perez-Jaramillo J, Cordovez V, Tracanna V, de Hollander M, Ruiz-Buck D, Mendes LW, van Ijcken WFJ, Gomez-Exposito R, Elsayed SS, Mohanraju P, Arifah A, van der Oost J, Paulson JN, Mendes R, van Wezel GP, Medema MH, Raaijmakers JM. 2019. Pathogen-induced activation of disease-suppressive functions in the endophytic root microbiome. Science 366:606–612. doi:10.1126/science.aaw9285. 31672892

[29] Chater KF, Biró S, Lee KJ, Palmer T, Schrempf H. 2010. The complex extracellular biology of *Streptomyces*. FEMS Microbiol Rev 34:171–198. doi:10.1111/j.1574-6976.2009.00206.x. 20088961

[30] Xu Z, Shao J, Li B, Yan X, Shen Q, Zhang R. 2013. Contribution of bacillomycin D in *Bacillus amyloliquefaciens* SQR9 to antifungal activity and biofilm formation. Appl Environ Microbiol 79:808–815. doi:10.1128/AEM.02645-12. 23160135 PMC3568560

[31] Pieterse CM, Zamioudis C, Berendsen RL, Weller DM, Van Wees SC, Bakker PA. 2014. Induced systemic resistance by beneficial microbes. Annu Rev Phytopathol 52:347–375. doi:10.1146/annurev-phyto-082712-102340. 24906124

[32] Tahir HAS, Gu Q, Wu H, Raza W, Safdar A, Huang Z, Rajer FU, Gao X. 2017. Effect of volatile compounds produced by *Ralstonia solanacearum* on plant growth promoting and systemic resistance inducing potential of *Bacillus volatiles*. BMC Plant Biol 17:133. doi:10.1186/s12870-017-1083-6. 28768498 PMC5541429

[33] Edgar RC. 2010. Search and clustering orders of magnitude faster than BLAST. Bioinformatics 26:2460–2461. doi:10.1093/bioinformatics/btq461. 20709691

[34] Delgado-Baquerizo M, Oliverio AM, Brewer TE, Benavent-González A, Eldridge DJ, Bardgett RD, Maestre FT, Singh BK, Fierer N. 2018. A global atlas of the dominant bacteria found in soil. Science 359:320–325. doi:10.1126/science.aap9516. 29348236

[35] Kidane D, Murphy D, Sweasy J. 2014. Accumulation of abasic sites induces genomic instability in normal human gastric epithelial cells during *Helicobacter pylori* infection. Oncogenesis 3:e128. doi:10.1038/oncsis.2014.42. 25417725 PMC4259965

[36] Meira LB, Bugni JM, Green SL, Lee C-W, Pang B, Borenshtein D, Rickman BH, Rogers AB, Moroski-Erkul CA, McFaline JL, Schauer DB, Dedon PC, Fox JG, Samson LD. 2008. DNA damage induced by chronic inflammation contributes to colon carcinogenesis in mice. J Clin Invest 118:2516–2525. doi:10.1172/JCI35073. 18521188 PMC2423313

[37] Rodriguez-R LM, Gunturu S, Harvey WT, Rosselló-Mora R, Tiedje JM, Cole JR, Konstantinidis KT. 2018. The Microbial Genomes Atlas (MiGA) webserver: taxonomic and gene diversity analysis of Archaea and Bacteria at the whole genome level. Nucleic Acids Res 46:W282–W288. doi:10.1093/nar/gky467. 29905870 PMC6031002

[38] Hu J, Wei Z, Friman V-P, Gu S-h, Wang X-f, Eisenhauer N, Yang T-j, Ma J, Shen Q-r, Xu Y-c, Jousset A. 2016. Probiotic diversity enhances rhizosphere microbiome function and plant disease suppression. mBio 7:e01790-16. doi:10.1128/mBio.01790-16. 27965449 PMC5156302

[39] Ji P, Wilson M. 2002. Assessment of the importance of similarity in carbon source utilization profiles between the biological control agent and the pathogen in biological control of bacterial speck of tomato. Appl Environ Microbiol 68:4383–4389. doi:10.1128/AEM.68.9.4383-4389.2002. 12200291 PMC124063

[40] Tans-Kersten J, Huang H, Allen C. 2001. *Ralstonia solanacearum* needs motility for invasive virulence on tomato. J Bacteriol 183:3597–3605. doi:10.1128/JB.183.12.3597-3605.2001. 11371523 PMC95236

[41] Hamady M, Knight R. 2009. Microbial community profiling for human microbiome projects: tools, techniques, and challenges. Genome Res 19:1141–1152. doi:10.1101/gr.085464.108. 19383763 PMC3776646

[42] Turnbaugh PJ, Hamady M, Yatsunenko T, Cantarel BL, Duncan A, Ley RE, Sogin ML, Jones WJ, Roe BA, Affourtit JP, Egholm M, Henrissat B, Heath AC, Knight R, Gordon JI. 2009. A core gut microbiome in obese and lean twins. Nature 457:480–484. doi:10.1038/nature07540. 19043404 PMC2677729

[43] Jiao S, Xu Y, Zhang J, Hao X, Lu Y. 2019. Core microbiota in agricultural soils and their potential associations with nutrient cycling. mSystems 4:e00313-18. doi:10.1128/mSystems.00313-18. 30944882 PMC6435817

[44] Tiedje JM, Asuming-Brempong S, Nüsslein K, Marsh TL, Flynn SJ. 1999. Opening the black box of soil microbial diversity. Appl Soil Ecol 13:109–122. doi:10.1016/S0929-1393(99)00026-8.

[45] Bardgett RD, Van Der Putten WH. 2014. Belowground biodiversity and ecosystem functioning. Nature 515:505–511. doi:10.1038/nature13855. 25428498

[46] Barberán A, Velazquez HC, Jones S, Fierer N. 2017. Hiding in plain sight: mining bacterial species records for phenotypic trait information. mSphere 2:e00237-17. doi:10.1128/mSphere.00237-17. 28776041 PMC5541158

[47] Santoyo G, Pacheco CH, Salmerón JH, León RH. 2017. The role of abiotic factors modulating the plant-microbe-soil interactions: toward sustainable agriculture. A review. Span J Agric Res 15:e03R01. doi:10.5424/sjar/2017151-9990.

[48] Liang Y, Xiao X, Nuccio EE, Yuan M, Zhang N, Xue K, Cohan FM, Zhou J, Sun B. 2020. Differentiation strategies of soil rare and abundant microbial taxa in response to changing climatic regimes. Environ Microbiol 22:1327–1340. doi:10.1111/1462-2920.14945. 32067386

[49] Sun B, Wang F, Jiang Y, Li Y, Dong Z, Li Z, Zhang XX. 2014. A long‐term field experiment of soil transplantation demonstrating the role of contemporary geographic separation in shaping soil microbial community structure. Ecol Evol 4:1073–1087. doi:10.1002/ece3.1006. 24772284 PMC3997323

[50] Liang Y, Jiang Y, Wang F, Wen C, Deng Y, Xue K, Qin Y, Yang Y, Wu L, Zhou J, Sun B. 2015. Long-term soil transplant simulating climate change with latitude significantly alters microbial temporal turnover. ISME J 9:2561–2572. doi:10.1038/ismej.2015.78. 25989371 PMC4817637

[51] Shi W, Li M, Wei G, Tian R, Li C, Wang B, Lin R, Shi C, Chi X, Zhou B, Gao Z. 2019. The occurrence of potato common scab correlates with the community composition and function of the geocaulosphere soil microbiome. Microbiome 7:14. doi:10.1186/s40168-019-0629-2. 30709420 PMC6359780

[52] Yang H, Li J, Xiao Y, Gu Y, Liu H, Liang Y, Liu X, Hu J, Meng D, Yin H. 2017. An integrated insight into the relationship between soil microbial community and tobacco bacterial wilt disease. Front Microbiol 8:2179. doi:10.3389/fmicb.2017.02179. 29163453 PMC5681905

[53] Bentley SD, Chater KF, Cerdeño-Tárraga AM, Challis GL, Thomson NR, James KD, Harris DE, Quail MA, Kieser H, Harper D, Bateman A, Brown S, Chandra G, Chen CW, Collins M, Cronin A, Fraser A, Goble A, Hidalgo J, Hornsby T, Howarth S, Huang CH, Kieser T, Larke L, Murphy L, Oliver K, O’Neil S, Rabbinowitsch E, Rajandream MA, Rutherford K, Rutter S, Seeger K, Saunders D, Sharp S, Squares R, Squares S, Taylor K, Warren T, Wietzorrek A, Woodward J, Barrell BG, Parkhill J, Hopwood DA. 2002. Complete genome sequence of the model actinomycete *Streptomyces coelicolor* A3(2). Nature 417:141–147. doi:10.1038/417141a. 12000953

[54] Reichenbach H. 2001. *Myxobacteria*, producers of novel bioactive substances. J Ind Microbiol Biotechnol 27:149–156. doi:10.1038/sj.jim.7000025. 11780785

[55] Alm RA, Lahiri SD. 2020. Narrow-spectrum antibacterial agents—benefits and challenges. Antibiotics 9:418. doi:10.3390/antibiotics9070418. PMC7400354 32708925

[56] Mojgani N. 2017. Bacteriocin-producing rhizosphere bacteria and their potential as a biocontrol agent, p 165–181. *In* Mehnaz S (ed), Rhizotrophs: plant growth promotion to bioremediation. Springer Singapore, Singapore.

[57] Rea MC, Dobson A, O’Sullivan O, Crispie F, Fouhy F, Cotter PD, Shanahan F, Kiely B, Hill C, Ross RP. 2011. Effect of broad-and narrow-spectrum antimicrobials on *Clostridium difficile* and microbial diversity in a model of the distal colon. Proc Natl Acad Sci USA 108:4639–4644. doi:10.1073/pnas.1001224107. 20616009 PMC3063588

[58] Lomeli BK, Galbraith H, Schettler J, Saviolakis GA, El-Amin W, Osborn B, Ravel J, Hazleton K, Lozupone CA, Evans RJ, Bell SJ, Ochsner UA, Jarvis TC, Baqar S, Janjic N. 2019. Multiple-ascending-dose phase 1 clinical study of the safety, tolerability, and pharmacokinetics of CRS3123, a narrow-spectrum agent with minimal disruption of normal gut microbiota. Antimicrob Agents Chemother 64:e01395-19. doi:10.1128/AAC.01395-19. 31685472 PMC7187627

[59] Clatworthy AE, Pierson E, Hung DT. 2007. Targeting virulence: a new paradigm for antimicrobial therapy. Nat Chem Biol 3:541–548. doi:10.1038/nchembio.2007.24. 17710100

[60] Macho AP. 2016. Subversion of plant cellular functions by bacterial type‐III effectors: beyond suppression of immunity. New Phytol 210:51–57. doi:10.1111/nph.13605. 26306858

[61] Yao J, Allen C. 2006. Chemotaxis is required for virulence and competitive fitness of the bacterial wilt pathogen *Ralstonia solanacearum*. J Bacteriol 188:3697–3708. doi:10.1128/JB.188.10.3697-3708.2006. 16672623 PMC1482862

[62] Jung B, Park J, Kim N, Li T, Kim S, Bartley LE, Kim J, Kim I, Kang Y, Yun K, Choi Y, Lee H-H, Ji S, Lee KS, Kim BY, Shon JC, Kim WC, Liu K-H, Yoon D, Kim S, Seo Y-S, Lee J. 2018. Cooperative interactions between seed-borne bacterial and air-borne fungal pathogens on rice. Nat Commun 9:31. doi:10.1038/s41467-017-02430-2. 29295978 PMC5750236

[63] Wang X, Li G-H, Zou C-G, Ji X-L, Liu T, Zhao P-J, Liang L-M, Xu J-P, An Z-Q, Zheng X, Qin Y-K, Tian M-Q, Xu Y-Y, Ma Y-C, Yu Z-F, Huang X-W, Liu S-Q, Niu X-M, Yang J-K, Huang Y, Zhang K-Q. 2014. Bacteria can mobilize nematode-trapping fungi to kill nematodes. Nat Commun 5:5776. doi:10.1038/ncomms6776. 25514608 PMC4275587

[64] Partida-Martinez LP, Hertweck C. 2005. Pathogenic fungus harbours endosymbiotic bacteria for toxin production. Nature 437:884–888. doi:10.1038/nature03997. 16208371

[65] Caporaso JG, Kuczynski J, Stombaugh J, Bittinger K, Bushman FD, Costello EK, Fierer N, Pena AG, Goodrich JK, Gordon JI, Huttley GA, Kelley ST, Knights D, Koenig JE, Ley RE, Lozupone CA, McDonald D, Muegge BD, Pirrung M, Reeder J, Sevinsky JR, Turnbaugh PJ, Walters WA, Widmann J, Yatsunenko T, Zaneveld J, Knight R. 2010. QIIME allows analysis of high-throughput community sequencing data. Nat Methods 7:335–336. doi:10.1038/nmeth.f.303. 20383131 PMC3156573

[66] Edgar RC. 2013. UPARSE: highly accurate OTU sequences from microbial amplicon reads. Nat Methods 10:996–998. doi:10.1038/nmeth.2604. 23955772

[67] DeSantis TZ, Hugenholtz P, Larsen N, Rojas M, Brodie EL, Keller K, Huber T, Dalevi D, Hu P, Andersen GL. 2006. Greengenes, a chimera-checked 16S rRNA gene database and workbench compatible with ARB. Appl Environ Microbiol 72:5069–5072. doi:10.1128/AEM.03006-05. 16820507 PMC1489311

[68] Price MN, Dehal PS, Arkin AP. 2009. FastTree: computing large minimum evolution trees with profiles instead of a distance matrix. Mol Biol Evol 26:1641–1650. doi:10.1093/molbev/msp077. 19377059 PMC2693737

[69] Jari Oksanen FGB, Friendly M, Kindt R, Legendre P, McGlinn D, Minchin PR, O’Hara RB, Simpson GL, Solymos P, Henry M, Stevens H, Szoecs E, Wagner H. 2015. vegan: Community Ecology Package. R package version 2.10.

[70] Wickham H. 2016. ggplot2: elegant graphics for data analysis. Springer, New York, NY.

[71] Rudis B, Bolker B, Schulz J, Kothari A, Sidi J. 2017. ggalt: extra coordinate systems,’Geoms’, statistical transformations, scales and fonts for ‘ggplot2’. R package version 0.4.0.

[72] Dhariwal A, Chong J, Habib S, King IL, Agellon LB, Xia J. 2017. MicrobiomeAnalyst: a web-based tool for comprehensive statistical, visual and meta-analysis of microbiome data. Nucleic Acids Res 45:W180–W188. doi:10.1093/nar/gkx295. 28449106 PMC5570177

[73] Parks DH, Tyson GW, Hugenholtz P, Beiko RG. 2014. STAMP: statistical analysis of taxonomic and functional profiles. Bioinformatics 30:3123–3124. doi:10.1093/bioinformatics/btu494. 25061070 PMC4609014

[74] Letunic I, Bork P. 2019. Interactive Tree Of Life (iTOL) v4: recent updates and new developments. Nucleic Acids Res 47:W256–W259. doi:10.1093/nar/gkz239. 30931475 PMC6602468

[75] Chen S, Zhou Y, Chen Y, Gu J. 2018. fastp: an ultra-fast all-in-one FASTQ preprocessor. Bioinformatics 34:i884–i890. doi:10.1093/bioinformatics/bty560. 30423086 PMC6129281

[76] Wingett SW, Andrews S. 2018. FastQ Screen: a tool for multi-genome mapping and quality control. F1000Res 7:1338. doi:10.12688/f1000research.15931.2. 30254741 PMC6124377

[77] Tamames J, Puente-Sanchez F. 2018. SqueezeMeta, a highly portable, fully automatic metagenomic analysis pipeline. Front Microbiol 9:3349. doi:10.3389/fmicb.2018.03349. 30733714 PMC6353838

[78] Li D, Liu C-M, Luo R, Sadakane K, Lam T-W. 2015. MEGAHIT: an ultra-fast single-node solution for large and complex metagenomics assembly via succinct de Bruijn graph. Bioinformatics 31:1674–1676. doi:10.1093/bioinformatics/btv033. 25609793

[79] Schmieder R, Edwards R. 2011. Quality control and preprocessing of metagenomic datasets. Bioinformatics 27:863–864. doi:10.1093/bioinformatics/btr026. 21278185 PMC3051327

[80] Wang Q, Garrity GM, Tiedje JM, Cole JR. 2007. Naive Bayesian classifier for rapid assignment of rRNA sequences into the new bacterial taxonomy. Appl Environ Microbiol 73:5261–5267. doi:10.1128/AEM.00062-07. 17586664 PMC1950982

[81] Hyatt D, Chen G-L, LoCascio PF, Land ML, Larimer FW, Hauser LJ. 2010. Prodigal: prokaryotic gene recognition and translation initiation site identification. BMC Bioinformatics 11:119. doi:10.1186/1471-2105-11-119. 20211023 PMC2848648

[82] Clark K, Karsch-Mizrachi I, Lipman DJ, Ostell J, Sayers EW. 2016. GenBank. Nucleic Acids Res 44:D67–D72. doi:10.1093/nar/gkv1276. 26590407 PMC4702903

[83] Kanehisa M, Goto S. 2000. KEGG: kyoto encyclopedia of genes and genomes. Nucleic Acids Res 28:27–30. doi:10.1093/nar/28.1.27. 10592173 PMC102409

[84] Buchfink B, Xie C, Huson DH. 2015. Fast and sensitive protein alignment using DIAMOND. Nat Methods 12:59–60. doi:10.1038/nmeth.3176. 25402007

[85] Langmead B, Salzberg SL. 2012. Fast gapped-read alignment with Bowtie 2. Nat Methods 9:357–359. doi:10.1038/nmeth.1923. 22388286 PMC3322381

[86] Wu Y-W, Simmons BA, Singer SW. 2016. MaxBin 2.0: an automated binning algorithm to recover genomes from multiple metagenomic datasets. Bioinformatics 32:605–607. doi:10.1093/bioinformatics/btv638. 26515820

[87] Kang DD, Li F, Kirton E, Thomas A, Egan R, An H, Wang Z. 2019. MetaBAT 2: an adaptive binning algorithm for robust and efficient genome reconstruction from metagenome assemblies. PeerJ 7:e7359. doi:10.7717/peerj.7359. 31388474 PMC6662567

[88] Sieber CM, Probst AJ, Sharrar A, Thomas BC, Hess M, Tringe SG, Banfield JF. 2018. Recovery of genomes from metagenomes via a dereplication, aggregation and scoring strategy. Nat Microbiol 3:836–843. doi:10.1038/s41564-018-0171-1. 29807988 PMC6786971

[89] Parks DH, Imelfort M, Skennerton CT, Hugenholtz P, Tyson GW. 2015. CheckM: assessing the quality of microbial genomes recovered from isolates, single cells, and metagenomes. Genome Res 25:1043–1055. doi:10.1101/gr.186072.114. 25977477 PMC4484387

[90] Ye Y, Doak TG. 2009. A parsimony approach to biological pathway reconstruction/inference for genomes and metagenomes. PLoS Comput Biol 5:e1000465. doi:10.1371/journal.pcbi.1000465. 19680427 PMC2714467

[91] Douglas GM, Maffei VJ, Zaneveld JR, Yurgel SN, Brown JR, Taylor CM, Huttenhower C, Langille MG. 2020. PICRUSt2 for prediction of metagenome functions. Nat Biotechnol 38:685–688. doi:10.1038/s41587-020-0548-6. 32483366 PMC7365738

[92] Blin K, Shaw S, Steinke K, Villebro R, Ziemert N, Lee SY, Medema MH, Weber T. 2019. antiSMASH 5.0: updates to the secondary metabolite genome mining pipeline. Nucleic Acids Res 47:W81–W87. doi:10.1093/nar/gkz310. 31032519 PMC6602434

[93] Sun H, Zhou N, Wang H, Huang DF, Lang ZH. 2017. Processing and targeting of proteins derived from polyprotein with 2A and LP4/2A as peptide linkers in a maize expression system. PLoS One 12:e0174804. doi:10.1371/journal.pone.0174804. 28358924 PMC5373624

[94] Jacobs JM, Babujee L, Meng F, Milling A, Allen C. 2012. The in planta transcriptome of *Ralstonia solanacearum*: conserved physiological and virulence strategies during bacterial wilt of tomato. mBio 3:e00114-12. doi:10.1128/mBio.00114-12. 22807564 PMC3413399

[95] Reasoner DJ, Geldreich EE. 1985. A new medium for the enumeration and subculture of bacteria from potable water. Appl Environ Microbiol 49:1–7. doi:10.1128/aem.49.1.1-7.1985. 3883894 PMC238333

[96] Roberts D, Denny T, Schell M. 1988. Cloning of the egl gene of *Pseudomonas solanacearum* and analysis of its role in phytopathogenicity. J Bacteriol 170:1445–1451. doi:10.1128/jb.170.4.1445-1451.1988. 2832363 PMC210987

[97] Boucher CA, Barberis PA, Demery DA. 1985. Transposon mutagenesis of *Pseudomonas solanacearum*: isolation of Tn5-induced avirulent mutants. Microbiology 131:2449–2457. doi:10.1099/00221287-131-9-2449.

[98] Song S. 2017. The effect and mechanism of biocontrol strain GZ-34 against Ralstonia solanacearum. Master’s thesis. South China Agricultural University, Guangzhou, China.

