# Supplementary figures and images for: Precision Probiotics in Agroecosystems: Multiple Strategies of Native Soil Microbiotas for Conquering the Competitor Ralstonia solanacearum
Source: mSystems. 2022 Apr 26;7(3):e01159-21. doi: 10.1128/msystems.01159-21 (PMC9239239; doi:10.1128/msystems.01159-21)

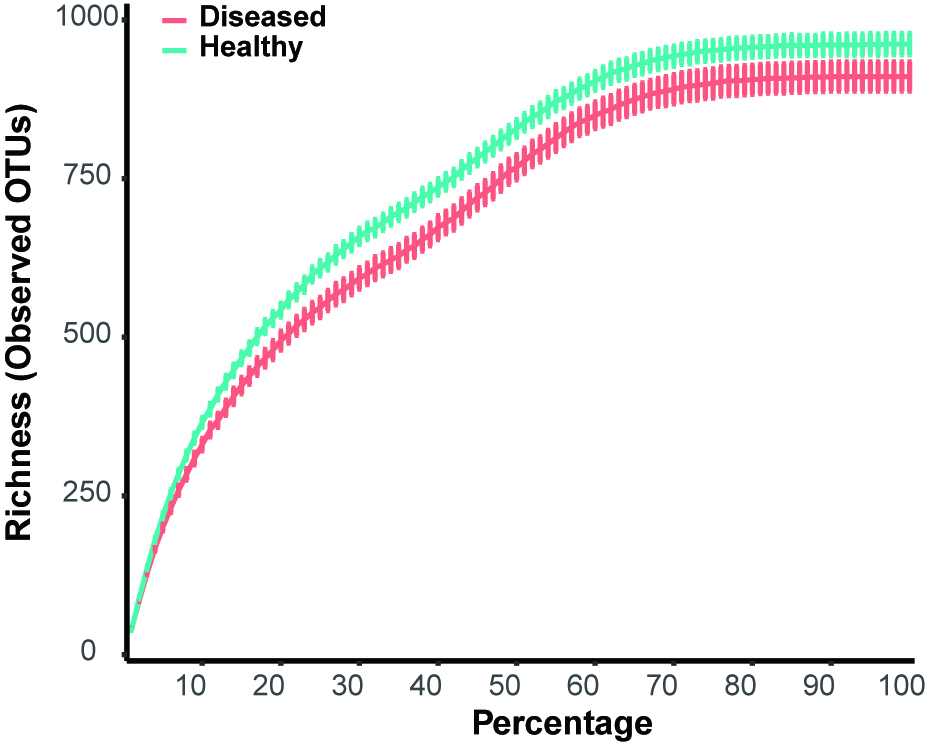

Supplement: FIG S1 [file msystems.01159-21-s0004.tif]

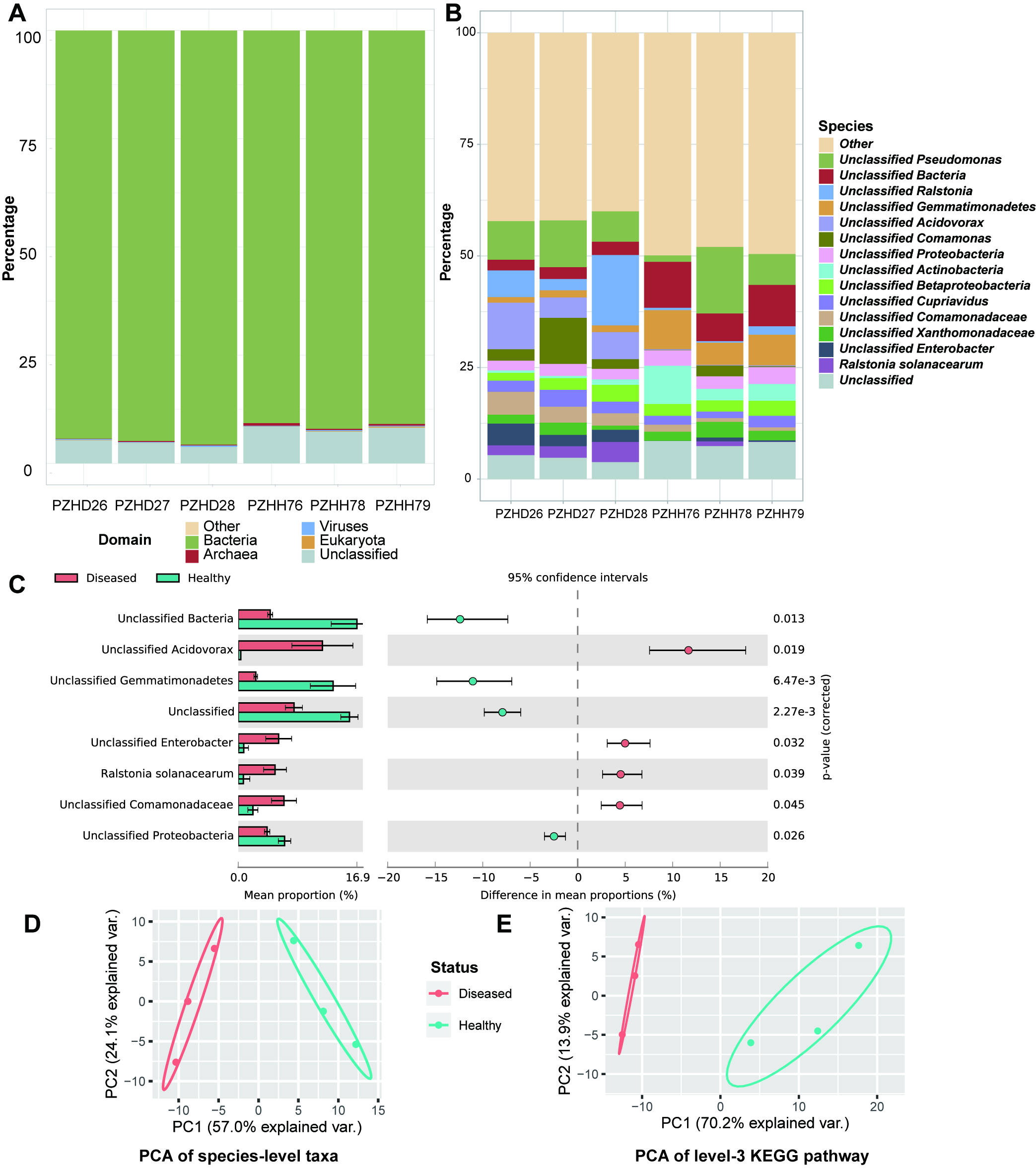

Supplement: FIG S2 [file msystems.01159-21-s0005.tif]

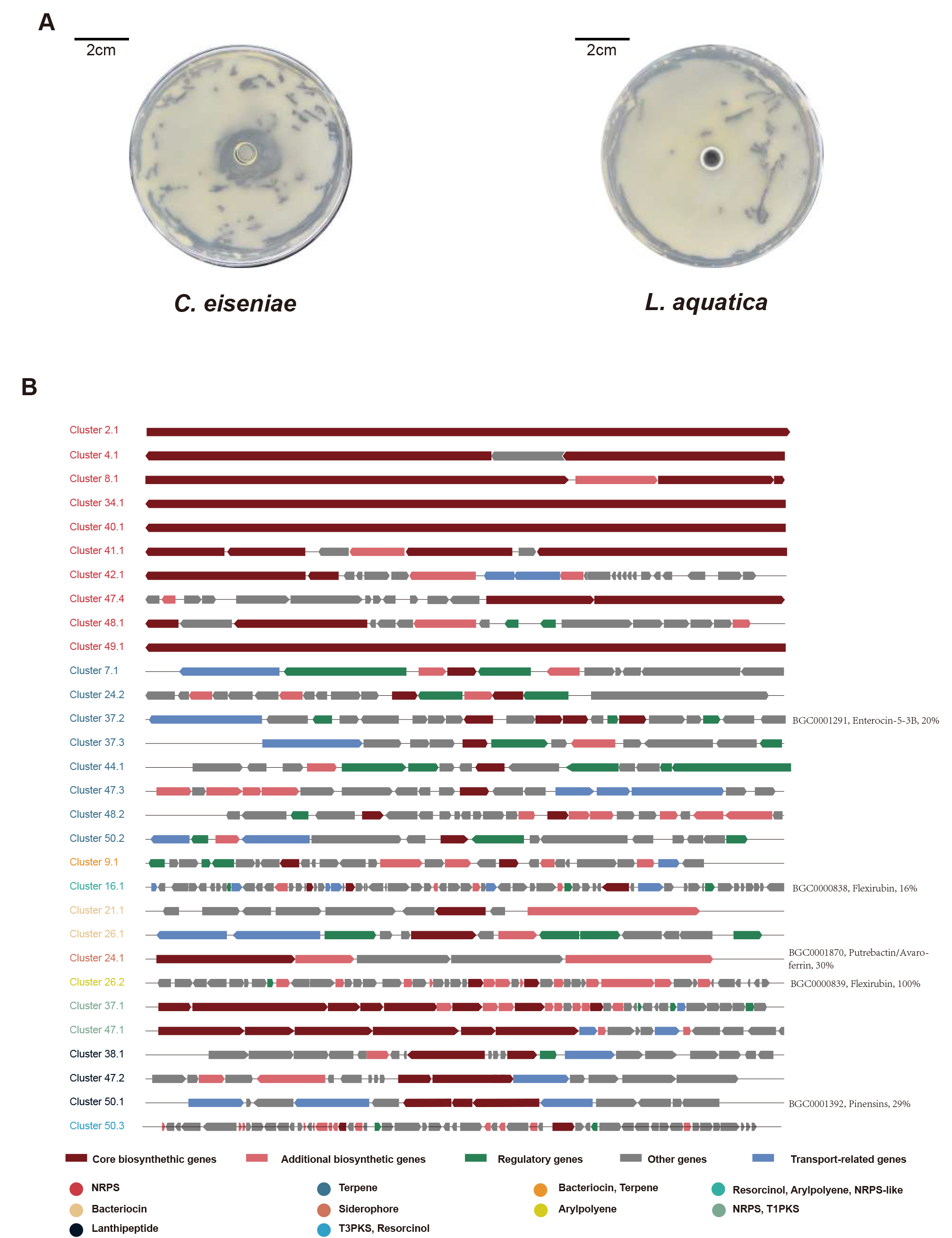

Supplement: FIG S4 [file msystems.01159-21-s0007.tif]

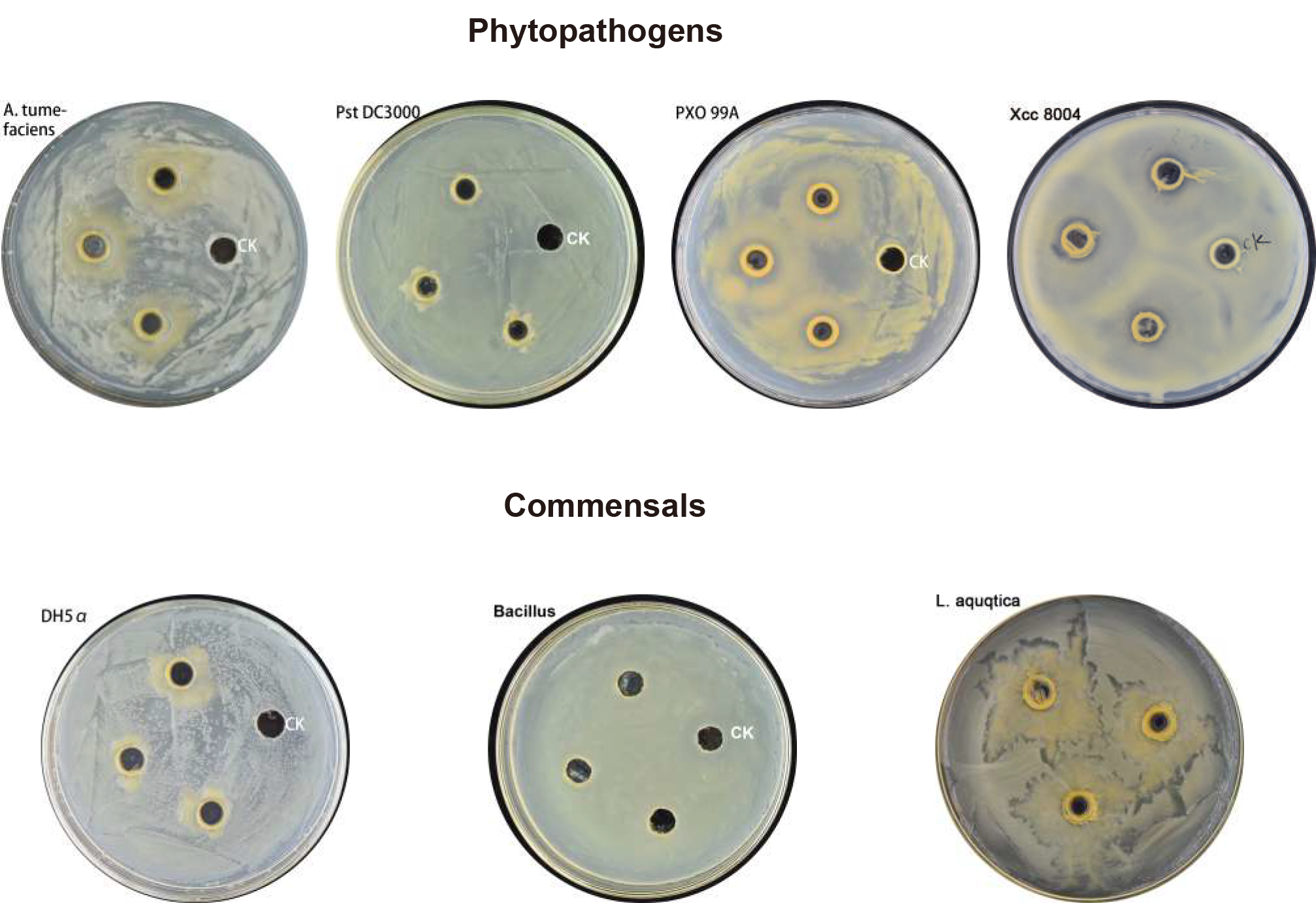

Supplement: FIG S5 [file msystems.01159-21-s0008.tif]

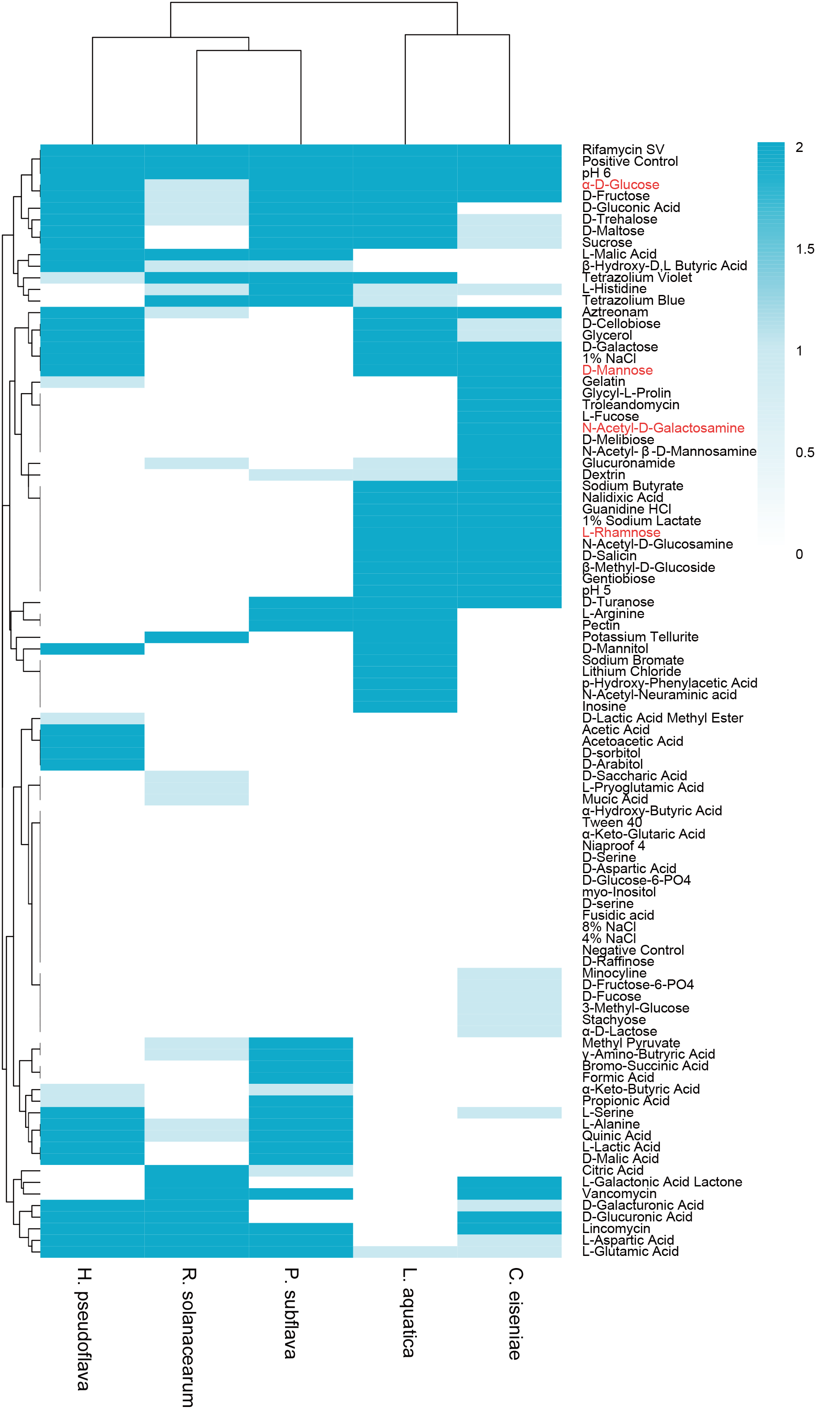

Supplement: FIG S6 [file msystems.01159-21-s0009.tif]
